# The Entomotoxic Fungal Lectin *Marasmius oreades* Agglutinin Disrupts the Midgut Epithelium of Colorado Potato Beetle Larvae

**DOI:** 10.1021/acs.jafc.5c16986

**Published:** 2026-02-17

**Authors:** Primož Žigon, Urban Bogataj, Sergej Praček, Tjaša Peternel, Polona Mrak, Maja Ivana Smodiš Škerl, Márta Ladányi, Katarina Karničar, Marko Fonović, Anastasija Panevska, Matej Skočaj, Dušan Turk, Nada Žnidaršič, Jaka Razinger, Jerica Sabotič

**Affiliations:** a Plant Protection Department, 54768 Agricultural Institute of Slovenia, Ljubljana 1000, Slovenia; b Department of Biology, Biotechnical Faculty, University of Ljubljana, Ljubljana 1000, Slovenia; c Department of Biotechnology, Jožef Stefan Institute, Ljubljana 1000, Slovenia; d Animal Production Department, 54768 Agricultural Institute of Slovenia, Ljubljana 1000, Slovenia; e Department of Applied Statistics, Institute of Mathematics and Basic Science, Hungarian University of Agriculture and Life Sciences (MATE), Budapest 1118, Hungary; f Department of Biochemistry and Molecular and Structural Biology, Jožef Stefan Institute, Ljubljana 1000, Slovenia; g Centre of Excellence for Integrated Approaches in Chemistry and Biology of Proteins, Ljubljana 1000, Slovenia

**Keywords:** biopesticide, entomotoxicity, European honeybee (Apis mellifera), fungal lectin, Leptinotarsa decemlineata, gut epithelium

## Abstract

Fungal lectins, with their specific glycan-binding activity, represent promising biopesticide candidates. This study investigates the potential of six fungal lectins to control Colorado potato beetle (CPB) larvae (*Leptinotarsa decemlineata*). *In vitro* feeding trials with recombinant lectins were performed to evaluate their individual toxicity. *Marasmius oreades* agglutinin (MOA) caused significantly higher mortality than the other lectins, whereas *Coprinopsis cinerea* lectin 2 (CCL2) and *Aleuria aurantia* lectin (AAL) completely inhibited weight gain. Functional analysis of MOA mutants revealed that both carbohydrate-binding and proteolytic domains are essential for toxicity. MOA binds to midgut glycoproteins in CPB larvae, disrupting midgut epithelium. Histology and ultramicroscopy showed that MOA causes loss of apicobasal polarity and detachment of basal lamina. No acute toxicity of lectins against adult European honeybees (*Apis mellifera*) was found; however, significant sublethal effects on larval development were observed. MOA shows promise as a selective bioinsecticide for sustainable CPB control with minimal nontarget effects.

## Graphical abstract

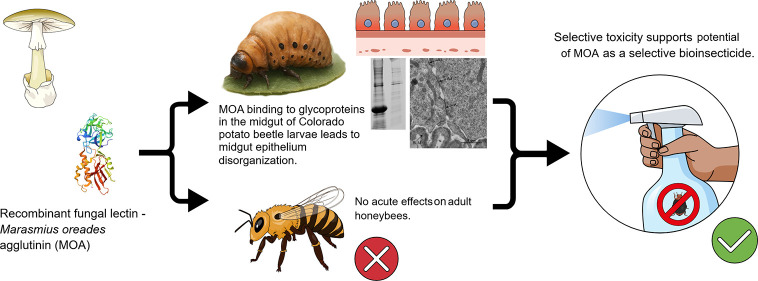

## Introduction

Plants respond to herbivorous insect attacks by producing specific proteins that target the insect digestive system, disrupting nutrient assimilation, enzymatic activity, and gut integrity. These proteins can alter the physiology and behavior of herbivorous insects, decreasing their ability to feed and reproduce.^1,2^ However, the effects of plants on herbivore development or mortality are often relatively small or even nonexistent, as insects overcome plant defenses through metabolic adaptations. Chemical insecticides have therefore been traditionally used to protect crops from pests; however, their use is costly and environmentally damaging, and thus naturally occurring and more sustainable options are needed.^3^ Insecticidal proteins are produced not only by plants but also by many other organisms and have potential for the development of environmentally friendly pest control strategies. Compared to synthetic insecticides, they have the advantage of more specific effects, rapid decomposition, and lower toxicity for nontarget organisms. In particular, the use of biopesticides based on insecticidal proteins or the incorporation of insecticidal genes into crops offers enormous improvements in pest control.^1,4^

Insecticidal proteins differ in their origins and effects on target organisms. Higher fungi are a rich source of these proteins and their coding genes.^5–7^ Research suggests that among fungal proteins, lectins are primarily responsible for insecticidal activity and thus have potential for pest control.^8–11^ The insecticidal effects of lectins are mainly due to their specific binding to carbohydrates in the insect’s gut, which disrupts nutrient absorption and digestive function, leading to decreased growth, development, and survival.^12,13^ Most research has targeted flies, aphids, lepidopteran caterpillars, and nematodes, with limited focus on beetles. Notable findings include the entomotoxic effects of *Xerocomus chrysenteron* lectin (XCL) and *Rhizoctonia solani* agglutinin (RSA) on *Drosophila melanogaster* and pea aphid (*Acrythosiphon pisum*).^14,15^ RSA has also been shown to be toxic against cotton leafworm (*Spodoptera littoralis*) larvae.^14^ Feeding trials using artificial diet showed that *Sclerotinia sclerotiorum* agglutinin (SSA) causes high mortality in pea aphids.^16^ *Sclerotium rolfsii* lectin (SRL; actinoporin-type lectin), which has significant toxicity against the cotton leafworm *Spodoptera litura*,^17^ also confers resistance against the sucking and chewing insects *S. litura* and *Aphis gossypii* in cotton plants expressing SRL.^18^ *Marasmius oreades* agglutinin (MOA) yields almost complete resistance against the beet cyst eelworm *Heterodera schachtii* in MOA-transformed *Arabidopsis* plants.^19^ In addition, nematotoxicity has been observed in some fruiting body lectins, including *Aleuria aurantia* lectin (AAL), *Coprinopsis cinerea* lectin 2 (CCL2), *Coprinopsis cinerea* galectin 2 (CGL2), and MOA.^20–23^ MOA is a chimeric beta-trefoil lectin with a C-terminal calcium-dependent cysteine protease domain and an N-terminal carbohydrate-binding domain that specifically binds to terminal α1–3-linked galactose.^24,25^ MOA exhibits toxicity against the model nematode *Caenorhabditis elegans* that depends on binding to glycosphingolipids via its lectin domain and on the proteolytic activity of its C-terminal catalytic domain.^21^ The toxicity of *C. cinerea* galectin 3 (CGL3)^20^ and cocaprin 1 (CCP1)^26^ against insects or nematodes has not yet been demonstrated.

The Colorado potato beetle (CPB) is considered one of the most important potato pests, as its destructive feeding behavior leads to losses in potato yield and quality, which ultimately affects the profitability of potato production.^27^ Controlling the damage caused by this pest is challenging due to its rapid reproduction and resistance to pesticides, as well as the need for sustainable and environmentally friendly control methods.^28^ Lectins from various origins have been studied for their potential to disrupt the gut physiology of CPB when ingested, thereby impairing nutrient absorption and ultimately leading to starvation or death of the insect.^5,29^ A plant lectin known as Gleheda, isolated from ground ivy (*Glechoma hederacea*), was the first with confirmed insecticidal activity against CPB larvae.^30^ To date, only one study has investigated the potential of fungal extracts against CPB.^8^ A study on extracts of the basidiomycete *Clitocybe nebularis* showed that lectins with different glycan-binding affinities act synergistically and lead to greater insecticidal efficacy than that of pure extracts of individual lectins. A crude extract containing different lactose-binding lectins decreased larval weight gain to a greater extent than that of pure lectins alone. Of the pure lectins tested in this study, only *C. nebularis* lectin (CNL) exerted a significant antinutritional effect on CPB larvae.^8^ In addition to lectins, fungal protease inhibitors have been considered as potential insecticidal agents against CPB. Both families of Mycocypins, clitocypins, and macrocypins affect larval growth and development by inhibiting specific digestive proteases without triggering adaptive responses at the transcriptional level in the larval gut.^31,32^

The evaluation of potential side effects on beneficial insects is an important step in the development of early stage biopesticide candidates to ensure the long-term success of innovative pest control strategies.^33^ For example, honeybees (*Apis mellifera* L.) play a crucial role in pollination and food production, and thus minimizing any side effects of pesticides is essential.^34^

In this study, we investigated the potential of selected lectins (Table 1) with different glycan-binding specificities (MOA, AAL, CGL2, CGL3, CCL2, and CCP1) to control CPB larvae. We evaluated the individual toxicities of these lectins with *in vitro* feeding trials and recombinant lectins. We further assessed the lectin MOA for its median lethal concentration (LC_50_) and mechanism of action in the CPB midgut, as well as molecular response of the larvae during feeding, to confirm its potential as a bioinsecticide. Additionally, we assessed the potential toxicity of lectins on *A. mellifera,* an established model organism that fulfils important (agro)­ecological functions.

**Table 1:** Overview of the Fungal Proteins Used for Toxicity Bioassays

Name	Origin	UniLectin3D Fold (PDB code)	Carbohydrate-binding specificity
*Aleuria aurantia* lectin (AAL)	*Aleuria aurantia*/Ascomycota	β-propeller (1OFZ)	Terminal α-Fuc^43^
*Coprinopsis cinerea* galectin 2 (CGL2)	*Coprinopsis cinerea*/Basidiomycota	Galectin (1UL9)	Gal-β1,4-Glc; Gal-β1,4-GlcNAc;Gal-β1,3-GalNAc; Gal-β1,4-Fuc^39^
*Coprinopsis cinerea* galectin 3 (CGL3)	*Coprinopsis cinerea*/Basidiomycota	Galectin (2R0F)	GalNAcβ1–4GlcNAc^40^
*Coprinopsis cinerea* lectin 2 (CCL2)	*Coprinopsis cinerea*/Basidiomycota	β-trefoil (2LIE)	Gal-β1,4(Fuc-α1,3)-GlcNAc;GlcNAc-β1,4(Fuc-α1,3)-GlcNAc^37^
Cocaprin 1 (CCP1)	*Coprinopsis cinerea*/Basidiomycota	β-trefoil (7ZNX)	Unknown, functions also as cysteine and aspartic protease inhibitor^26^
*Marasmius oreades* agglutinin (MOA)	*Marasmius oreades*/Basidiomycota	β-trefoil chimeric (2IHO)	Gal-α1,3-Gal/GalNAc-β ^44,45^
*Marasmius oreades* agglutinin mutant MOA Q46AW138A	*Marasmius oreades*/Basidiomycota	β-trefoil chimeric	No binding^21^
*Marasmius oreades* agglutinin mutant MOA C215A	*Marasmius oreades*/Basidiomycota	β-trefoil chimeric	Gal-α1,3-Gal/GalNAc-β^21^
*Marasmius oreades* agglutinin mutant MOA ΔC	*Marasmius oreades*/Basidiomycota	β-trefoil	Gal-α1,3-Gal/GalNAc-β^21^

## Materials and Methods

### Preparation of Recombinant Fungal Proteins

Recombinant fungal proteins (Table 1) were produced in a bacterial expression system. Ligation-independent cloning (LIC) protocols were used to clone the coding sequences of the lectins into the expression vector pMCSG7, which adds a His-tag to the N-terminus that can be removed by a Tobacco Etch Virus (TEV) protease cleavage site:^35^ *Aleuria aurantia* lectin (AAL, UniProt ID: P18891 ^36^), *Coprinopsis cinerea* lectin 2 (CCL2, UniProt ID: B3GA02 ^37^), *Marasmius oreades* agglutinin (MOA, UniProt ID: Q8X123 ^38^), *Coprinopsis* galectin 2 (CGL2, UniProt ID: Q9P4R8 ^39^), *Coprinopsis* galectin 3 (CGL3, UniProt ID: Q206Z5,^40^ and Cocaprin 1 (CCP1 Uniprot ID: A8PCJ3 ^26^). To generate MOA mutants (Table 1), PCR site-directed mutagenesis was performed using KOD Hot Start DNA Polymerase (Sigma-Aldrich) and the expression plasmid pMCSG7-MOA as template, followed by Dpn1 digestion to obtain vectors with mutant MOA versions.^41^ The recombinant proteins were produced in the *Escherichia coli* host strains BL21­(DE3) or BL21­(DE3)-RIL (Agilent Technologies). Bacteria were grown in ZYM-5052 autoinduction medium for 4 h at 37 °C followed by 20 h at 18 °C^42^ and then harvested by centrifugation and lysed by sonication in buffer A (30 mM Tris-HCl, pH 7.5, 400 mM NaCl) with 1 mg/mL lysozyme and cOmplete EDTA-free Protease Inhibitor Cocktail (Roche). The lysate was clarified by centrifugation, and proteins were purified using a two-step protocol: (1) NiNTA (HisTrap FF 5 mL column, Cytiva) using buffer A with 10 mM imidazole for binding and 300 mM imidazole for elution and (2) Size Exclusion Chromatography (HiPrep 26/60 Sephacryl S-200 HR or S-100 HR column, Cytiva) with buffer A. Fractions containing the protein of interest were collected, concentrated, dialyzed against phosphate-buffered saline (PBS; 137 mM NaCl, 2.7 mM KCl, 10 mM Na_2_HPO_4_, 1.8 mM KH_2_PO_4_, pH 7.4) and filter-sterilized.

### Toxicity Bioassays on CPB Larvae

Protein toxicity against CPB larvae was determined with a leaf disc bioassay.^7^ CPB larvae originated from a laboratory colony established in June 2021 from adults collected from local potato fields in central Slovenia and kept on potted potato plants under greenhouse conditions at the Agricultural Institute of Slovenia. Randomly selected larvae of developmental stages L2–L3 were fed potato leaf discs treated with lectins and protease inhibitors. Leaf discs (d = 14 mm) were freshly excised from potato leaves (variety ˈKIS Kokraˈ) and soaked in solutions of different lectins for 5 min. Lectin solutions were prepared at 1 mg/mL in PBS and for simple sugar competition, preincubation with 100 mM methyl α-D-galactopyranoside (Biosynth, Slovakia) was used. Leaf discs treated with buffer (PBS) served as the negative control, and those treated with a 0.1% dilution of Laser plus insecticide (a.i. spinosad, 48% w/w; Corteva AgriSciences, USA) served as the positive control. Leaf discs were drained and transferred to six-well plates.

A single larva was placed on a treated leaf disc in each well to begin exposure. Each treatment contained three plates, giving a total of 18 individuals per treatment for survival monitoring. The experiment was conducted for 5 days in an incubation chamber at 22 ± 1 °C and 77% relative humidity and a photoperiod of 14:10 h light:dark. The bioassay was repeated twice independently, giving a total of 36 larvae per treatment. Supplementary tests were conducted using the same experimental procedure to assess the effect of the reference bioinsecticide Novodor FC (*Bacillus thuringiensis* subsp. *tenebrionis* str. NB 176, 2% m/m, Biofa GmbH, Germany) at 0.1% concentration on the mortality and weight gain of CPB larvae. Larval mortality and leaf disc consumption were recorded daily during the 5 days of the experiment. A visual assessment of consumed leaf disc area was made using a five-point scale (0–25, 25–50, 50–75, and 75–100% leaf disc consumed), and freshly treated potato leaf discs were offered when more than 90% of the leaf disc was consumed. The larvae were weighed individually at the beginning and after 5 days. The weight gain was calculated for each larva that survived the 5 day bioassay. To obtain LC_50_, the insecticidal activities of five MOA concentrations (0–2 mg/mL) were tested. The LC_50_ bioassay lasted only 48 h due to the high mortality rate at higher MOA concentrations (1 and 2 mg/mL), using the same procedure as described above.

### Glycolipid Analysis

Polar lipids were extracted from CPB larvae or larval gut tissue using a modified protocol.^46^ Briefly, wet tissue (1 g) was homogenized in cold deionized water (dH_2_O; 3 mL), cold methanol (8 mL), and cold chloroform (4 mL), with vortexing after each addition. The suspension was shaken overnight at room temperature and centrifuged at 2,500 g for 15 min. The first supernatant was collected and stored at 4 °C. The remaining pellet was re-extracted with the same solvent ratios, and the second supernatant was combined with the first supernatant. Then dH_2_O was added to adjust the solvent (dH_2_O:methanol:chloroform) ratio from 3:8:4 to 5.6:8:4, promoting phase separation upon centrifugation (15 min, 2,500 g, room temperature). The upper (polar) phase was collected, dried using a rotary evaporator, and rehydrated in 3.2 mL of the original 3:8:4 solvent mixture using glass beads and vortexing. The lipid suspension was dried under nitrogen and resuspended in the same solvent system to a final concentration of 10 mg/mL.

For surface plasmon resonance (SPR) analysis, the extracted polar lipids were mixed at a 1:1 molar ratio with a mixture of 1-palmitoyl-2-oleoyl-sn-glycero-3-phosphocholine (POPC):cholesterol and then dried to form lipid films. Cholesterol was added to this lipid mixture to obtain more stable lipid vesicles. In the case of gut-derived extracts, only POPC was used. Lipid films were hydrated in PBS to a final concentration of 5 mg/mL and vortexed to form multilamellar vesicles. These were extruded through a 100 nm polycarbonate membrane at 60 °C to generate large unilamellar vesicles. SPR binding studies were performed using a Biacore T200 system with an L1 sensor chip, and PBS was used as a running buffer. Large unilamellar vesicles were immobilized on the sensor chip, and 10 μM MOA was injected at 10 μL/min with an association and dissociation time of 60 and 290 s or with an association and dissociation time of 120 and 230 s, respectively. The second flow cell was used for sample analysis, and the first flow cell served as a reference to correct for nonspecific binding. The chip was regenerated using 0.5% SDS, 40 mM octyl-β-glucoside, and 30% ethanol (10 μL/min). All experiments were conducted at 25 °C and analyzed using BIAevaluation software (version 3.2.1).

### Preparation of CPB Midgut Protein Extracts, MOA Affinity Chromatography, and Mass Spectrometry

L3 CPB larvae were transferred to an empty Petri dish and left for 3 days without a food source to allow them to empty their gut contents. The gut of each larva was then removed with tweezers, taking care to acquire as much midgut as possible. The gut sample obtained from 69 larvae (488.3 mg) was stored at – 80 °C. The frozen biological material was then ground to a fine powder in liquid nitrogen using a pestle and mortar. The ground material was transferred to two chilled 2 mL centrifuge tubes, and 350 μL extraction buffer (20 mM Tris-HCl, 0.4 M NaCl, 2 mM EDTA, 0.1% NP-40, pH 7.4) was added to each, vortexed briefly, and incubated on ice for 15 min. After incubation, the samples were centrifuged (5 min, 31,150 g, 4 °C), and the supernatant was stored in aliquots at – 80 °C or used immediately for affinity chromatography.

Recombinant MOA was immobilized to CNBr-activated Sepharose (GE Healthcare) according to the manufacturer’s instructions (45 mg of MOA was coupled to 2 g of lyophilized Sepharose). MOA-Sepharose (400 μL) was transferred to a Mobicol column (MoBiTec) and equilibrated in extraction buffer. An equal amount of crude protein extract from the midgut of CPB larvae (250 μL) was applied to MOA-Sepharose and to Sepharose as a control for nonspecific binding and incubated for 1 h at 4 °C with gentle mixing. After washing the unbound fractions with the extraction buffer, the bound proteins were eluted by boiling Sepharose in SDS-PAGE sample buffer for 10 min. They were then analyzed with precast “Invitrogen Novex Tris-Glycine Mini Protein Gels, 4–20%” and Coomassie Blue staining. Bands in the MOA-bound eluate were compared to those in the Sepharose-bound eluate. Selected individual bands, which were visible in the Coomassie-stained gel, present in the MOA-bound eluate, and absent from the Sepharose-bound eluate, were excised for further analysis (Figure S1).

Excised bands were subjected to in-gel trypsin digestion and identified by mass spectrometry using an Orbitrap Velos mass spectrometer coupled to a Proxeon Nano-LC HPLC unit (Thermo Fisher Scientific). The results were analyzed using MaxQuant proteomics software (version 2.0.3.0) and a Uniprot database. For potential target proteins whose subcellular location has not yet been experimentally determined, we used various online tools for prediction based on their similarity to other proteins or to characteristic sequences, such as signal peptides. We used the “WoLF PSORT: protein localization predictor”^47^ and “BUSCA: an integrative web server to predict subcellular localization of proteins”^48^ to determine multiple potential subcellular locations, “PredGPI: a GPI-anchor predictor”^49^ to determine membrane proteins present, and DeepTMHMM^50^ to determine protein topology. Potential glycosylation profiles were analyzed using online tools to determine potential O- and N-glycosylation sites, including NetOGlyc 4.0 ^51^ and NetNGlyc 1.0.^52^ MOA-affinity chromatography and subsequent mass spectrometry of potential molecular targets of MOA was performed twice.

### Analysis of the Glycoprotein Profile for Molecular Target Proteins

MOA and MOA Q46AW138A mutant were fluorescently labeled with the Atto647N Protein Labeling Kit (Sigma) according to the manufacturer’s protocol. The crude protein extracts from the guts of L3 CPB larvae were treated with PNGase F (New England Biolabs) and O-glycosidase with α2–3,6,8 neuraminidase bundle (New England Biolabs) according to the manufacturer’s protocols and analyzed on 10% SDS-PAGE along with the same amount (3 μL) of untreated extract and transferred to a nitrocellulose membrane. The membrane was incubated for 1 h at room temperature in binding buffer (50 mM Tris-HCl, pH 7.5, 150 mM NaCl, and 0.5% Tween 20). Subsequently, the MOA-bound proteins were detected with fluorescently labeled MOA or MOA nonglycan-binding mutant followed by five 5 min wash steps in binding buffer. The MOA-reactive bands were visualized by fluorescence detection (644/669 nm) using the ChemiDoc Imaging System (Bio-Rad).

### Transmission Electron and Light Microscopy

L3 larvae fed on control and MOA-treated (1 mg/mL) leaf discs from the MOA toxicity bioassays were used for microscopic analysis of the gut. Larvae were collected after 24 h of exposure and prepared for microscopy. Whole larvae (the anterior region or posterior abdominal segments were removed to enable the penetration of fixatives and resin) and isolated guts obtained by dissection were fixed and processed for light and transmission electron microscopy. All specimens were fixed in 2.5% glutaraldehyde and 2% formaldehyde in 0.1 M HEPES buffer and rinsed and postfixed in 1% OsO_4_ in the same buffer. After rinsing, the samples were dehydrated in ethanol and acetone, embedded in Agar 100 epoxy resin and prepared for sectioning. Semithin (0.5 μm) and ultrathin (70 nm) sections were prepared using an ultramicrotome (Reichert Ultracut S, Leica). Semithin sections were mounted onto glass slides, stained with Azure II–methylene blue, and imaged with a light microscope (AxioImager Z.1, Zeiss), equipped with a camera (AxioCam HRc, Zeiss) and AxioVision software (Zeiss). Ultrathin sections were mounted onto copper grids and contrasted with uranyl acetate and lead citrate. Imaging was performed with a transmission electron microscope (CM 100, Philips), equipped with an Orius SC200 camera and Digital Micrograph Suite software (Gatan). Micrographs were processed with FIJI software (ImageJ). The stitching of multiple contiguous micrographs to obtain images of larger areas was performed using the TrakEM2 plugin for FIJI. The image panels were prepared in Adobe Illustrator.

### Toxicity Bioassays on Honeybees

To test potential effects of lectins on nontarget organisms, we conducted toxicity bioassays on honeybee (*A. mellifera carnica*, Poll. 1879) adult workers and larvae. Worker bees were collected from a healthy honeybee colony and sorted into hoarding cages (plastic 3 dL cups). The cups were prepared before the experiment by making several 1–2 mm holes for ventilation and two larger holes for a syringe (5 mL), one on top for feeding and another on the side for the removal of dead bees. A saccharose solution (1:1) was added to the cups, which were placed overnight in an incubator at 27 °C and 60% humidity. The next day, the bees were deprived of food for 1.5–2 h, and then the test solutions were added: MOA, CGL2, AAL, CCL2, CCP1 (all 1 mg/mL), PBS (buffer), H_2_O (for negative control), and the toxic standard dimethoate (0.1 μg and 0.3 μg active ingredient per bee, reference material, Honeywell, for positive control). All groups were then monitored at three time points (24, 48, and 72 h), and the numbers of dead bees were recorded. The test was performed in accordance with a previous method.^53^ The limit test for MOA was performed according to OECD/OCDE 213,^54^ and 100 μg of active ingredient per bee was used to demonstrate that LC_50_ is greater than this value. The procedure included three replicate test groups for the test dose and the relevant controls, including the toxic standard.

The second trial was conducted on bee larvae. We performed a chronic test with artificial rearing of bee larvae in an incubator. Honeycombs containing young larvae (up to 24 h old) were collected from healthy honeybee colonies. The larvae were then transplanted onto preprepared sterile 48-well microtiter plates with plastic pots inserted, in accordance with previous methods.^53^ Larvae received the same treatments as adult bees. All groups were monitored for 5 days, and the numbers of dead larvae were recorded. On the last day of the experiment, surviving larvae were weighed.

### Statistical Analysis

CPB feeding rates and weight changes were analyzed using robust one-way ANOVAs (Brown-Forsythe tests) to account for potential violations of the homogeneity of variance assumption. Prior to analysis, transformations were applied to improve normality: feeding rate data in the screening tests of lectins against CBP larvae were transformed using a natural logarithm (ln), and weight change data in the recombinant MOA experiment were transformed using a square root. Normality of residuals was assessed by examining the absolute values of skewness and kurtosis; in all cases, these values were <1, indicating acceptable normality. When the Levene’s test indicated a violation of the homogeneity of variances (*p* < 0.05), pairwise comparisons were conducted using the Games-Howell post hoc test, which is robust to unequal variances. Larval weight data were analyzed in two ways. Initially, when the homogeneity of variances assumption was met (Levene’s test, *p* > 0.05), one-way ANOVA was performed. Following the removal of four outliers, the normality of residuals was confirmed (as described above). For a separate analysis of larval weights where the homogeneity of variance assumption was violated (Levene’s test, *p* < 0.05), robust one-way ANOVA (Brown-Forsythe test) was employed, followed by Games-Howell post hoc tests. The insecticidal activity of MOA on CPB was expressed as LC_50_, determined by nonlinear sigmoid curve fitting of MOA concentrations against relative mortality. The feeding rate of honeybees was measured at three time points (4, 24, and 48 h) in three trials of toxicity bioassays. Data were analyzed using one-way MANOVA. Subsequent univariate ANOVAs were performed with Bonferroni’s correction for multiple comparisons. Pairwise comparisons were then conducted using the Games-Howell post hoc test. Mortality data were analyzed using Kaplan–Meier survival analysis, and survival curves were compared using the log-rank test. Pairwise comparisons were performed with a Benjamini-Hochberg correction^55^ to control the false discovery rate. Cox proportional hazard ratios were calculated to demonstrate that experimental replicates did not result in statistically significantly different results. Statistical analysis was performed using R (version 4.4.1, R Core team, 2024) and the packages ‘survminer’^56^ and ‘survival’.^57^ Graphs were made in GraphPad Prism version 8.4.3 (GraphPad Software, San Diego, CA, USA).

## Results

### Toxicity Screening of Fungal Lectins Against CPB Larvae

Selected fungal lectins (Table 1) were produced as recombinant proteins in a bacterial expression system (Figure S2). Their insecticidal properties were evaluated in a feeding bioassay with treated, excised potato leaf discs. Except for CCL2, which did not exert any negative effects on CPB larval survival, all other lectins (MOA, AAL, CGL2, CGL3, and CCP1) and spinosad significantly decreased the survival rate (χ^2^(7) = 222.0; *P* < 0.001) during these 5-day bioassays. MOA decreased survival to the greatest extent and caused significantly higher mortality compared to the other lectins (except spinosad). Spinosad caused the fastest mortality: > 90% of the larvae were dead after 2 days. MOA caused 50% larval mortality after 2 days, and the mortality rate on the fourth day was 75% higher than that in the control group (Figure 1a and Figure S3).

**Figure 1:**
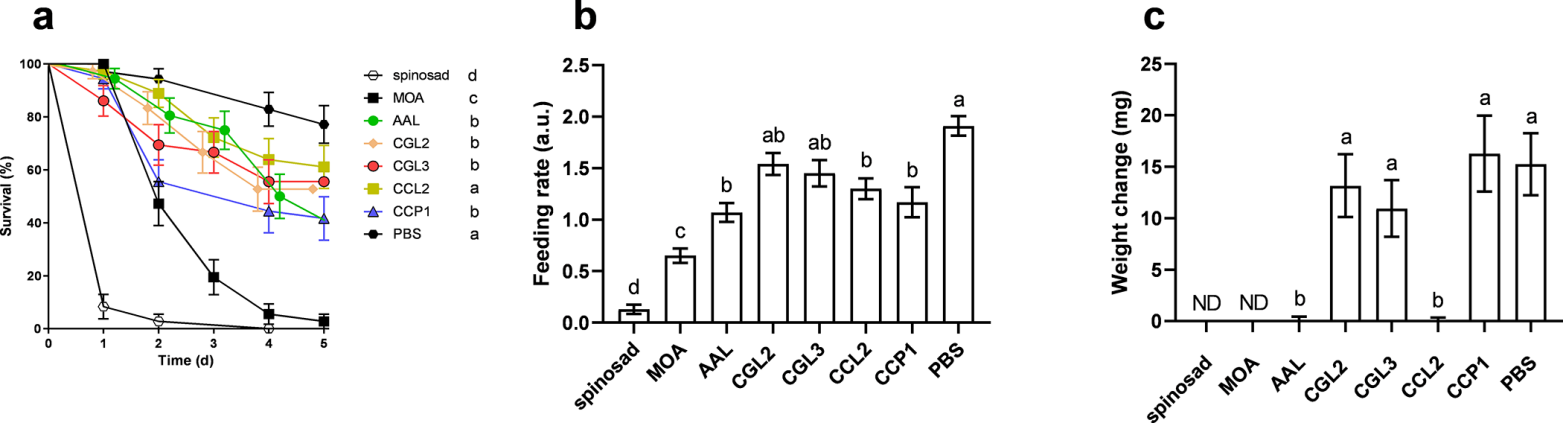
Feeding trials with Colorado potato beetle (CPB) larvae and lectins. Excised potato leaf discs were treated with lectins (MOA, AAL, CGL2, CGL3, CCL2, CCP1; all 1 mg/mL), spinosad (positive control), and phosphate-buffered saline (PBS; negative control). The feeding trials lasted for 5 days. (a) Survival curves, (b) feeding rates, and (c) weight changes of CPB larvae. Weight changes after spinosad and MOA treatment (labeled as ND) could not be evaluated because there were no live larvae after 5 days. Different lowercase letters above bars indicate significant differences (*P* < 0.05) between treatments, assessed with the Benjamini-Hochberg (a) and Games-Howell post hoc tests (b and c).

Feeding activity was significantly decreased when exposed to leaf discs treated with CCP1, CCL2, AAL, MOA, and spinosad (Brown-Forsythe (BF): F­(7, 216.95) = 29.11, *P* < 0.0001). MOA showed the strongest antifeeding effect. The feeding rate was approximately six times lower than that of the control larvae and comparable to the treatment with spinosad. Conversely, the antifeeding effects of CGL2 and CGL3 did not differ significantly, with feeding rates comparable to those of the control group (Figure 1b).

Regarding weight, larvae fed with leaves treated with CGL2, CGL3, CCP1 and PBS accumulated biomass at a faster rate compared to the larvae in other treatments (Figure 1c). Feeding on leaves treated with CCL2, AAL, and MOA resulted in a negative growth rate (BF: F­(5, 71.33) = 8.49, *P* < 0.0001), indicating sublethal effects in the form of starvation and possibly other physiological effects. The mean larval weight gain after PBS treatment was 15.2 ± 3.01 mg. After 5-day-lectin treatments, it ranged from 11 mg (CGL2) to 16.3 mg (CCP1) (Figure 1c). No weight gain was observed after treatments with CCL2 and AAL, indicating the entomotoxic effects of the accumulated lectins. After treatments with spinosad and MOA, no live individuals remained after 5 days. For comparison, we conducted supplementary trials using the bioinsecticide Novodor FC, which is based on *B. thuringiensis* subsp. *tenebrionis* (Figure S4). Larvae exposed to Novodor-FC-treated leaf discs exhibited a significantly lower survival rate compared to the control group (21.74 ± 8.60%; χ^2^(1) = 35.70, *P* < 0.0001), and their weight gain after 5 days was minimal (0.47 ± 1.59 mg; *t*(74) = 6.03, *P* < 0.0001). As MOA showed the strongest insecticidal activity on CPB larvae among the lectins tested (Figure 1), we next focused on investigating its mode of action and effects on the physiology of treated larvae.

### MOA Toxicity and Mode of Action

MOA at a concentration of 2 mg/mL resulted in 100% larval mortality within 48 h. The entomotoxic potential of MOA was confirmed, with an LC_50_ value of 0.67 mg/mL determined by sigmoid regression (95% confidence interval = 0.31–1.04 mg/mL; R^2^ = 0.74; Figure S5). The D’Agostino-Pearson test confirmed that the sigmoid dose–response model residuals followed a normal distribution (χ^2^= 1.87, *P* = 0.393).

### Carbohydrate Binding and Proteolytic Activity are Essential for the Toxicity of MOA Against CPB Larvae

To evaluate the mechanism of action of MOA toxicity, two mutants were produced that lack proteolytic activity (MOA C215A with an inactive proteolytic domain but the ability to form dimers and MOA ΔC with an always monomeric glycan-binding domain), and one mutant was produced that lacks carbohydrate-binding activity but can form dimers via its active proteolytic domain (MOA Q46AW138A) (Table S1). In addition, an inhibitory sugar, methyl-α-D-galactopyranoside (Me-α-Gal, 100 mM), which was expected to inhibit glycan binding by MOA, was used to mimic the inactive glycan-binding mutant. The insecticidal activity of the recombinant MOA mutants was investigated in a 5-day bioassay, and Kaplan–Meier survival analysis revealed a significant result (χ^2^(6) = 162; *P* < 0.001). Benjamini-Hochberg follow-up pairwise comparison showed that Me-α-Gal did not inhibit MOA toxicity, as treatment with both MOA and Me-α-Gal showed significant toxicity against CPB larvae. This was the only treatment that showed an insecticidal effect besides the MOA treatment used as a positive control. Inactivation of carbohydrate-binding activity (MOA Q46AW138A), removal of the proteolytic C-terminal domain (MOA ΔC), and inactivation of only the cysteine protease activity of the C-terminal domain (MOA C215A) all effectively abolished the entomotoxicity of MOA (Figure 2a). However, MOA Q46AW138A achieved this to a much lesser extent than MOA C215A (Figure 2c).

**Figure 2:**
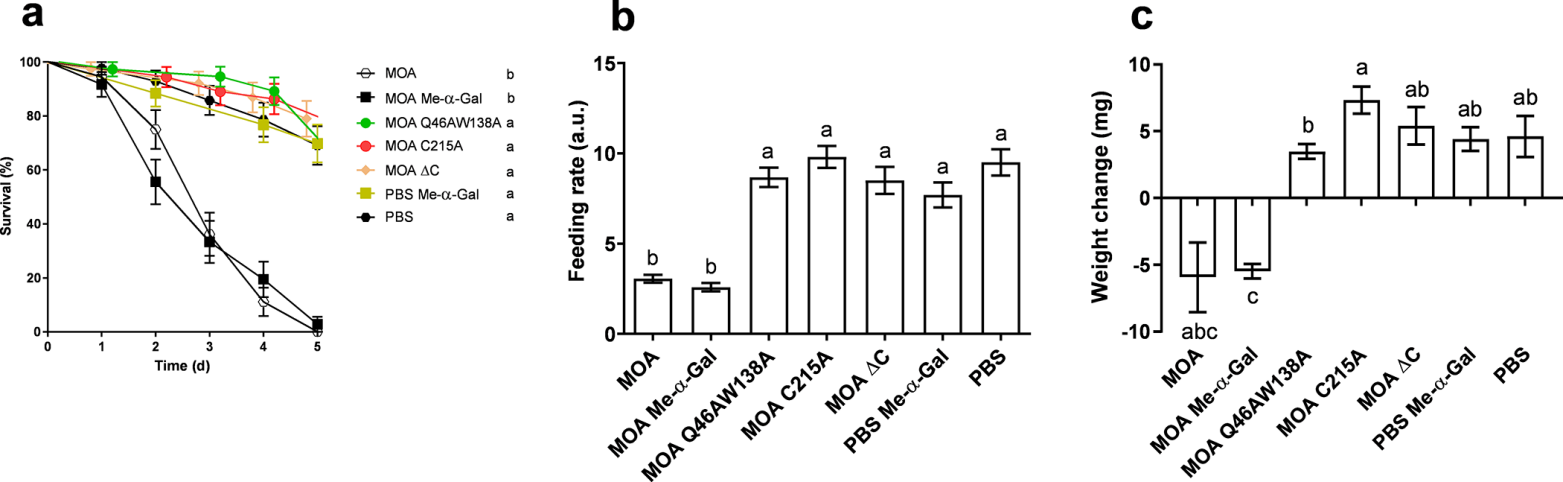
Feeding trials with Colorado potato beetle (CPB) larvae and *M. oreades* agglutinin (MOA). Excised potato leaf discs were treated with recombinant MOA (MOA, MOA Me-α-Gal, MOA Q46AW138A, MOA C215A, and MOA ΔC; all 0.5 mg/mL), phosphate-buffered saline (PBS) Me-α-Gal, and control (PBS). The feeding trials lasted for 5 days. (a) Survival curves, (b) feeding rates, and (c) weight changes of CPB larvae. Different lowercase letters above bars indicate significant differences (*P* < 0.05) between treatments, assessed with the Benjamini-Hochberg (a) and Games-Howell post hoc tests (b and c).

The treatments significantly affected the feeding rate (BF: F­(6, 180.87) = 27.36; *P* < 0.001). Games-Howell multiple comparisons test demonstrated that MOA and MOA Me-α-Gal significantly decreased the feeding rate, whereas other treatments did not differ significantly from the negative control (PBS) (Figure 2b). The treatments also significantly affected weight change (BF: F­(6, 11.37) = 5.79; *P* < 0.001). As demonstrated by Games-Howell multiple comparisons test, the only significant difference was between MOA Me-α-Gal and PBS (Figure 2c). Although MOA decreased the feeding rate the most, the small sample size (n = 3) and high standard deviation did not yield a statistically significant result.

### MOA Binds to Glycoproteins in the Midgut of CPB Larvae

We tested whether MOA binds to glycolipids present in the polar lipid extracts from either whole CPB larvae or only their gut, reconstituted into artificial lipid membranes. Although these extracts contained a fraction of glycolipids, no MOA binding was detected under the experimental conditions (Figure S6). Next, we evaluated whether MOA targets glycoproteins in CPB larval guts. We analyzed the binding of MOA to proteins in glycosidase-treated protein extracts of CPB larval guts and compared it with that of the carbohydrate-binding mutant MOA Q46AW138A. We confirmed binding to glycoproteins, which was abolished by PNGase F and O-glycosidase, which remove N- and O-glycans from glycoproteins, respectively (Figure 3). Some nonspecific binding of MOA and MOA Q46AW138A by the protease domain was expected; however, a few protein bands were identified that lose binding of MOA after glycosidase treatment and are not bound by the MOA mutant without carbohydrate-binding capability. Their apparent molecular masses had ranges of either 110–130 kDa or 70–80 kDa.

**Figure 3:**
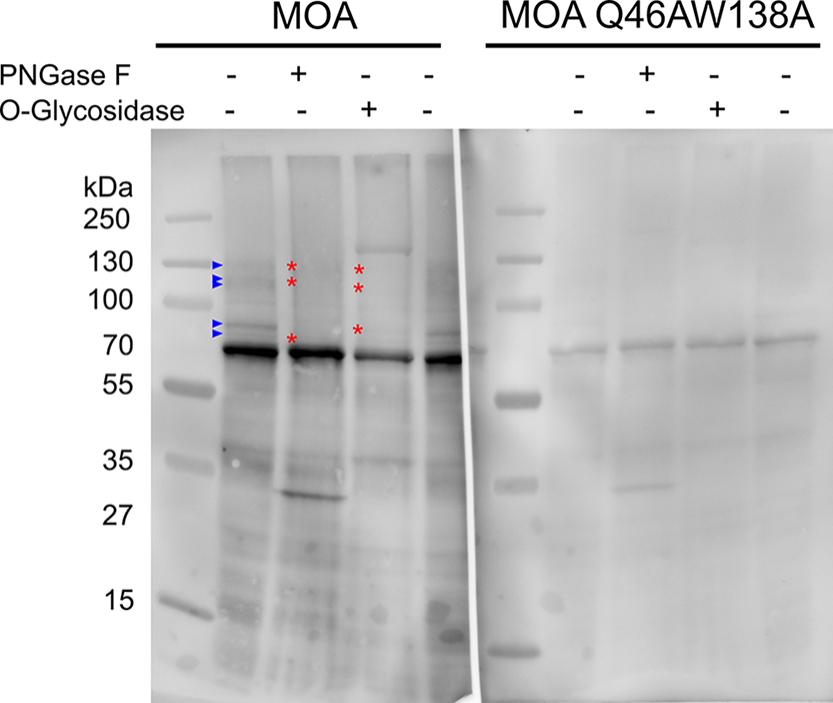
Analysis of the binding of *M. oreades* agglutinin (MOA) to glycoproteins in the gut extract of Colorado potato beetle (CPB) larvae. The crude protein extract from CPB larvae midguts was treated with PNGase F or O-glycosidase and analyzed on 10% SDS-PAGE. Western blot detection was performed using fluorescently labeled MOA (left) or MOA Q46AW138A (right). Blue arrowheads indicate protein bands that bound MOA in untreated control samples but are absent in glycosidase-treated samples with removed N- or O-glycans (indicated by red asterisks) and also did not bind the MOA Q46AW138A carbohydrate-binding mutant (absent in the right panel).

Furthermore, to determine the molecular targets of MOA, affinity chromatographic isolation of protein extracts from the midgut of CPB larvae was performed, and proteins bound to MOA and not to the Sepharose control were identified by peptide mass fingerprinting (Table 2 and Tables S2 and S3 and Figures S1 and S7). A likely MOA target is aminopeptidase, a glycoprotein localized to the cell membrane that potentially enables MOA endocytosis. This 112 kDa plasma membrane protein is involved in peptide processing, signaling, and the transport and recycling of membrane proteins via endocytosis.^58^ In addition, clathrin was identified as a potential MOA target and is located at the plasma membrane, where it plays a role in cellular protein transport and is essential for clathrin-mediated endocytosis.^59^ However, clathrin is not glycosylated and accumulates on the inner side of the membrane. Thus, it is probably not bound by the glycan-binding domain of MOA, but either indirectly via the actual MOA partner or via the protease domain of MOA. In addition to clathrin, several other intracellular potential molecular targets of the proteolytic activity of MOA have been identified, including proteins involved in cellular metabolism and stress responses (Table 2).

**Table 2:** Identification of Proteins that Interact with *M. oreades* Agglutinin (MOA) in the Gut Extracts of Colorado Potato Beetle (CPB) Larvae^a^

Protein ID	Protein name	MW (Da)	Unique peptides first	Unique peptides second	Molecular/biological function	Biological process	Subcellular location	Putative N-glycosylation sites	Putative O-glycosylation sites
V9PBG4	Cathepsin B	52,076	3	9	cysteine-type peptidase activity	proteolysis	extracellular, lysosome	4	9
E7CIZ1	Glycoside hydrolase family protein 48	73,245	17	8	glycosidase	carbohydrate metabolism, polysaccharide degradation	extracellular	0	2
A0A0A7ENR4	Carboxylic ester hydrolase	63,353	8	3	carboxylic ester hydrolase	lipid metabolism	extracellular	4	1
A0A290GAZ2	Yellow-x2	64,765	3	3	major royal jelly protein family	larval or pupal development	extracellular	5	20
**D9J2F5**	**Aminopeptidase**	**111,583**	**7**	**7**	**metalloaminopeptidase**	**proteolysis**	**cell membrane (GPI-anchor)**	**27**	**42**
A0A2D1QUF3	Clathrin heavy chain	192,798	12	8	structural molecule	intracellular protein transport, vesicle organization	membrane, cytoplasmic vesicle membrane	5	1
Q3I414	Cytochrome P450	58,729	6	19	oxidoreductase	P450-containing electron transport chain	endoplasmic reticulum membrane	1	1
U6BQX5	Heat shock protein 70a	71,101	21	10	chaperone	stress response	cytoplasm	6	1
A0A0E3ISE4	Actin	41,770	7	10	cytoskeleton molecule	actin filament organization	cytoplasm	1	2
V5QPM9	Heat shock protein 83	82,087	5	3	unfolded protein binding, chaperone	stress response	cytoplasm	4	7
A0A2D1QUE3	E1 ubiquitin-activating enzyme	116,638	2	3	ubiquitin-activating enzyme	DNA damage response, protein catabolic process	cytoplasm, nucleus	7	17
V5QQR2	Heat shock 60 kDa protein	61,265	10	8	chaperone	stress response	mitochondrial matrix	2	5
G9FQ75	Multifunctional fusion protein	63,280	2	4	oxidoreductase	proline metabolism	mitochondrial matrix	3	0

a MOA-affinity pull-down followed by mass spectrometry of bound proteins was performed twice independently. Only proteins that were detected in both replicates are listed. The detected peptides are indicated in the protein sequences in Figure S7. The complete list of interacting proteins in each replicate is provided in Tables S2 and S3. The putative primary target of the MOA glycan-binding domain, which is glycosylated and located at the plasma membrane, is indicated in bold, whereas the others are putative targets of the MOA proteolytic domain.

### MOA Exposure Leads to Midgut Epithelium Disorganization and Accumulation of Structurally Aberrant Cells

After 24 h of exposure to MOA, larval midgut epithelium was completely disorganized compared to that of control larvae. Conversely, the hindgut epithelium of treated larvae did not show any distinct modifications and was structurally similar to that of control larvae (Figure 4). The midgut epithelium of control larvae comprised a monolayer of mostly apicobasally polarized prismatic enterocytes. Small clusters of stem cells were scattered in the basal part of the epithelium between the enterocytes (Figure 4a). In contrast to control larvae, the midgut epithelium of MOA-treated larvae comprised several layers of cells that did not contain prismatic enterocytes or show structural characteristics of polarization. Small cells in the basal part of the epithelium displayed relatively intact histological structure and roughly resembled stem cells, whereas the cells above them gradually degraded toward the interior of the midgut lumen, which was filled with cell debris (Figure 4b). The hindgut epithelium in both control and MOA-treated larvae comprised a monolayer of dome-shaped cells that protruded basally into the hemocoel (Figure 4c,d).

**Figure 4:**
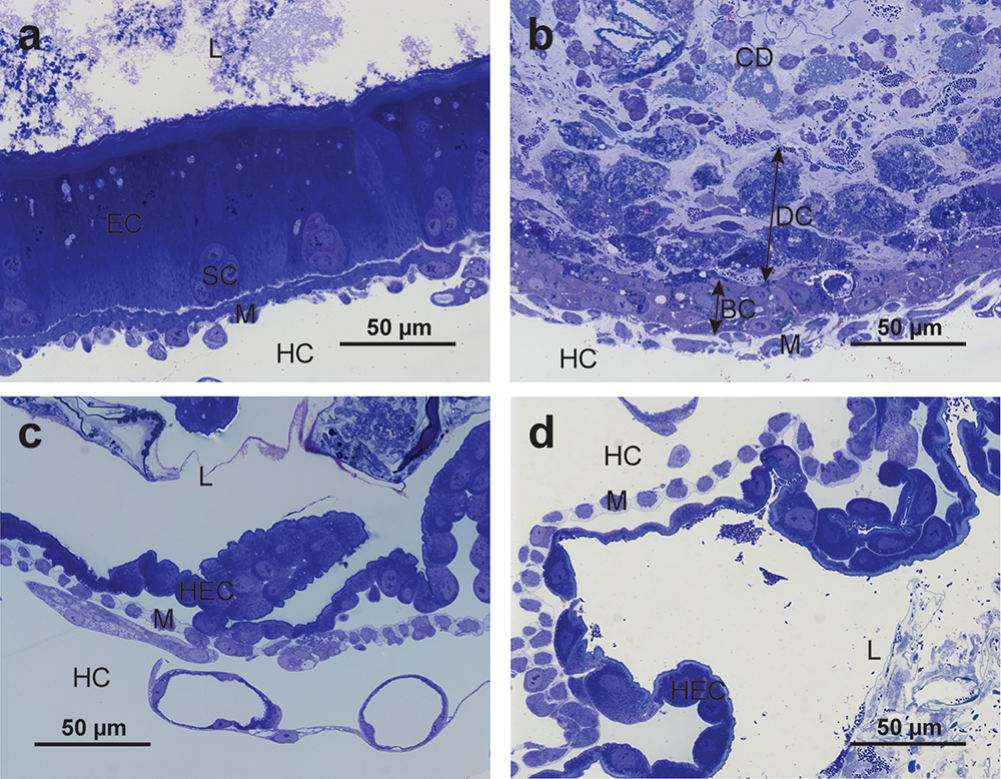
Histological structure of the gut epithelium of untreated and *M. oreades* agglutinin (MOA)-treated Colorado potato beetle (CPB) larvae. a) Control midgut epithelium consists of numerous enterocytes (EC) and clusters of stem cells (SC). b) MOA-treated midgut epithelium after 24 h consists of cells arranged in several layers. Basally located cells (BC) are small and roughly resemble the SCs of control larvae. Detached cells (DC) above have lost polarity and are disintegrating. The lumen is filled with cell debris (CD). c) Control hindgut epithelium with one layer of dome-shaped hindgut epithelial cells (HEC). d) MOA-treated hindgut epithelium after 24 h is structurally similar to control hindgut epithelium. L: gut lumen, HC: hemocoel, M: muscle.

At the ultrastructural level, one of the most conspicuous modifications in MOA-treated larvae was the loss of the apicobasal polarity of midgut cells (Figure 5). In control larvae, the epithelial cells of the midgut were clearly apicobasally polarized. Apically, they had a pronounced brush border of microvilli, and the basal plasma membrane formed an extensive basal labyrinth. In the subapical cytoplasm, abundant mitochondria were evident (Figure 5a). In MOA-treated larvae, the apicobasal polarity was distinctively lost. Some of the small basally located cells above the basal lamina showed limited structural characteristics of polarization, such as small clusters of microvilli at the apical surface. Above the small basal cells, larger detached isodiametric cells that completely lacked cell polarity were present. The detached cells gradually disintegrated toward the center of the midgut lumen, which was filled with debris of degraded midgut cells. Extensive intercellular spaces were apparent between the midgut cells of the treated larvae (Figure 5b).

**Figure 5:**
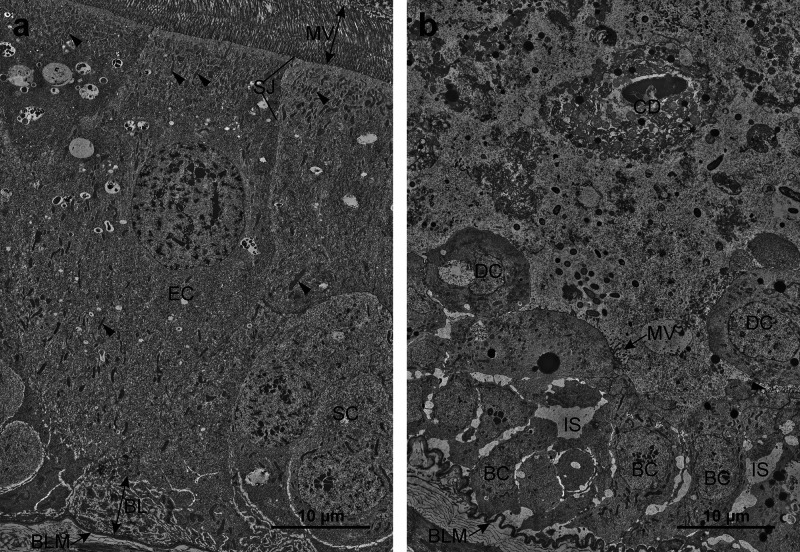
Ultrastructure of the midgut cells of untreated and *M. oreades* agglutinin (MOA)-treated Colorado potato beetle (CPB) larvae. a) Control midgut epithelium consists mainly of polarized enterocytes (EC) with a brush border of microvilli (MV) on the apical side and basal labyrinth (BL) on the basal side. Abundant mitochondria (arrowheads) are concentrated in the subapical region. Small clusters of stem cells (SC) are located between the enterocytes. b) MOA-treated midgut epithelium mostly does not exhibit apicobasal polarity. Some of the basally located cells (BC), which are above the basal lamina (BLM), have small clusters of microvilli (MV) on their apical surface. Large intercellular spaces (IS) are present between these cells. The detached cells (DC) above the basal cells completely lack apicobasal polarity. In some areas between the basal and detached cells, small clusters of microvilli are observed. The midgut lumen is filled with cell debris (CD).

In control larvae, the apical surface of midgut cells exhibited numerous long microvilli, which were of equal length and densely arranged at regular intervals. Neighboring midgut cells were subapically connected by pronounced septate junctions, followed basally by several adherens junctions. The profiles of the septate junctions were orientated in the apicobasal direction (Figure 6a). The basal plasma membrane of midgut cells was folded in a deep basal labyrinth. The basal lamina lining the basal side of the midgut epithelium was in close contact with the basal plasma membrane, and hemidesmosome-like junctions attached the midgut cells to the basal lamina (Figure 6b). In the midgut epithelium of MOA-treated larvae, the apical plasma membrane of the small basally located cells was mostly flat with only small clusters of irregular microvilli. Occasionally, the clusters of microvilli were found in small intercellular spaces between the small basal cells and larger detached cells above. The septate junctions were present in the subapical region, but their profiles were orientated in different directions (Figure 6c). Several adherens junctions were observed underneath the septate junctions, similarly as in control larvae. The basal plasma membrane in the midgut cells of MOA-treated larvae did not form a basal labyrinth. The basal lamina was separated from the basal plasma membrane by a narrow space, and hemidesmosome-like junctions were absent. The basal lamina was not apposed to the epithelium and displayed a wavy profile (Figure 6d).

**Figure 6:**
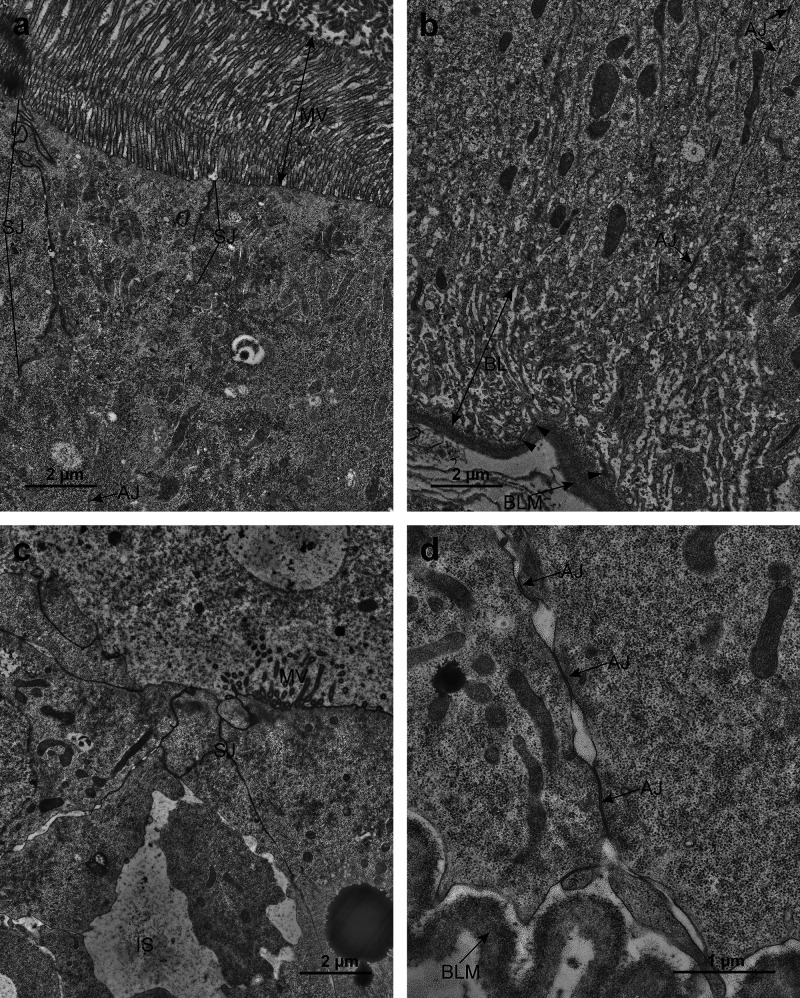
Ultrastructure of microvilli, cell junctions, and basal lamina in midgut cells of untreated and *M. oreades* agglutinin (MOA)-treated Colorado potato beetle (CPB) larvae. a) Midgut cells of control larvae have a brush border of long and densely arranged microvilli (MV) on the apical surface. Subapically, the profiles of septate junctions (SJ) are orientated in the apicobasal direction, and adherens junctions (AJ) are present underneath. b) Midgut cells of control larvae have an extensive basal labyrinth (BL) formed by basal plasma membrane. The basal lamina (BLM) is in close contact with the basal plasma membrane, and hemidesmosome-like junctions (arrowheads) connecting cells with lamina are evident. c) In MOA-treated larvae after 24 h, some of the basally located cells exhibit only small clusters of irregular microvilli (MV). The profiles of septate junctions (SJ) are orientated in different directions, with extensive intercellular spaces (IS) underneath. d) In MOA-treated larvae after 24 h, the basal plasma membrane is mostly flat and separated from the basal lamina (BLM). No hemidesmosome-like junctions are present. Numerous adherens junctions (AJ) are evident between neighboring cells.

Similarly, as observed at the histological level, the hindgut epithelium showed no discernible ultrastructural modifications between control and MOA-treated larvae. In both control and MOA-treated larvae, the hindgut cells were apically lined by a chitinous cuticle, exhibited distinct apical and basal plasma membrane labyrinths, and were laterally connected by a complex of adherens and extensive convoluted septate junctions (Figure 7).

**Figure 7:**
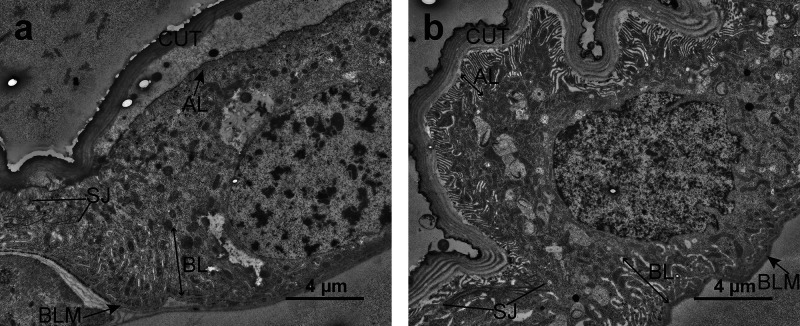
Ultrastructure of hindgut cells of untreated and *M. oreades* agglutinin (MOA)-treated Colorado potato beetle (CPB) larvae. a) Hindgut cells of control larvae are apically lined with cuticle (CUT). The apical and basal plasma membranes form the apical (AL) and basal (BL) labyrinths, respectively. Neighboring cells are laterally connected with adherens junctions and extensive convoluted septate junctions (SJ). b) Hindgut cells of MOA-treated larvae after 24 h show similar ultrastructure as that of control larvae. BLM: basal lamina.

### Toxicity Bioassays on Honeybees

MOA, AAL, CGL2, CCL2, and CCP1 were also tested for their entomotoxicity against the adults and larvae of honeybees (*A. mellifera*), a species that provides important pollination services in (agro)­ecosystems.

### Screening of Fungal Lectin Toxicity Against Adult Honeybees

The concentrations of fungal lectins in the acute toxicity assays with adult worker honeybees were the same as those in the CPB feeding bioassays. Kaplan–Meier survival analysis revealed a significant result of the treatments (χ^2^(9)=2365; *P* < 0.001). Benjamini-Hochberg pairwise comparison showed no differences between the tested lectins and negative control (PBS), indicating that the lectins were not toxic. The decreased survival of honeybees in the positive control (dimethoate) validates the screening test (Figure 8a). The block design repeated measures model for the feeding rate resulted in a significant overall result for time (Wilk’s lambda = 0.27, *P* < 0.001) and treatment effect (F­(9,112) = 2.99, *P* < 0.01) (Figure 8b).

**Figure 8:**
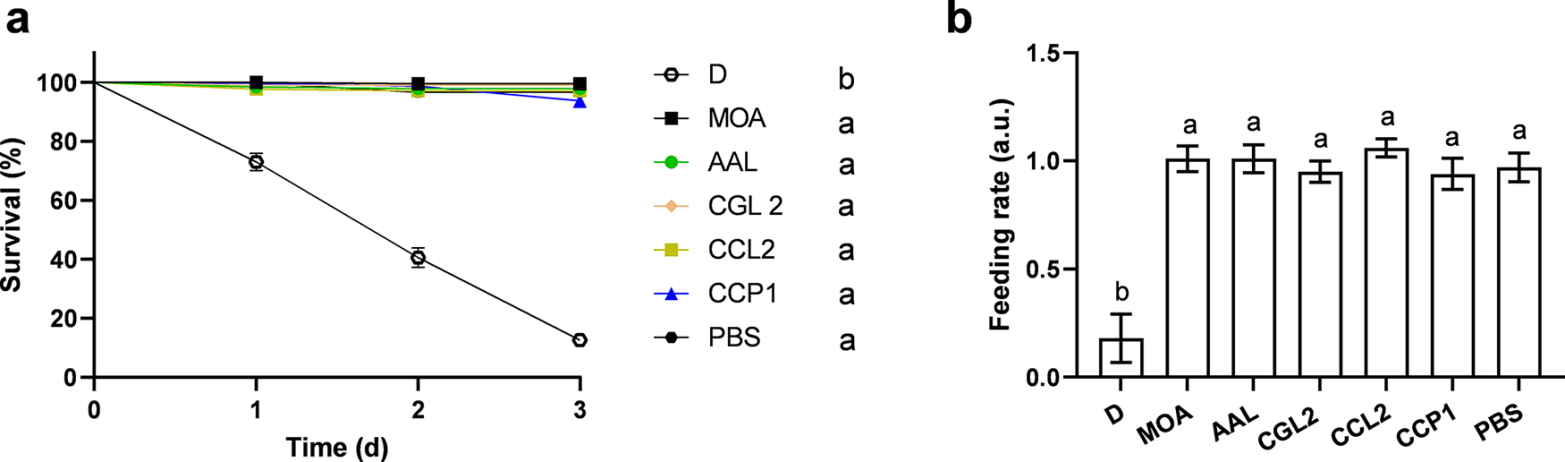
Feeding trials of adult *A. mellifera.* The following lectins were used: MOA, AAL, CGL2, CCL2, and CCP1 (all at 1 mg/mL). Dimethoate (D) and phosphate-buffered saline (PBS) were used as positive and negative controls, respectively). The trials lasted for 3 days. (a) Survival curves and (b) feeding rates of *A. mellifera*. Different lowercase letters indicate significant differences (*P* < 0.05) between treatments, assessed with the Benjamini-Hochberg (a) and Games-Howell post hoc tests (b).

Next, we tested higher MOA concentrations to further evaluate its entomotoxic potential. LC_50_ was calculated based on the trial with MOA at 1, 2, and 3 mg/mL, following OECD guidelines^54^ for the limit test. The LC_50_ values exceeded 100 μg a.i./bee; therefore, MOA can be classified as having low acute toxicity to honeybees, as confirmed by the limit test approach.

### Screening of Fungal Lectin Toxicity against Honeybee Larvae

Bioassays were conducted by artificially rearing bee larvae for 5 days with the lectins MOA, AAL, CGL2, CCL2, and CCP1 at the same concentrations as in the CPB feeding bioassays. Kaplan–Meier survival analysis revealed a significant overall difference among treatment groups (χ^2^(7) = 282, *P* < 0.001). This effect was attributable to the positive control (dimethoate), which exhibited a survival rate that significantly differed from that of the negative control (PBS). By contrast, none of the lectin treatments differed significantly from the negative control, as confirmed by the Benjamini-Hochberg pairwise comparisons (Figures S8a, S9). Conversely, the lectins significantly affected larval weight (Brown-Forsythe: F­(7,330.383) = 6.39; *P* < 0.001). The results of the pairwise comparisons according to the Games-Howell post hoc test are provided in Figure S8b. The lowest weight was found for MOA, CGL2, and CCL2; however, these differences did not significantly differ from the negative control (PBS) (Figure S8b).

## Discussion

Research into various substances of natural origin is making an important contribution to the development of alternative, nonchemical pest control methods. For example, fungi are a rich source of lectins with unique carbohydrate-binding specificities. These lectins participate in numerous physiological functions such as defense against fungivores, including insects. The insecticidal effects of lectins are not due to general cytotoxic action but rather specific lectin–carbohydrate interactions.^8,60^ This makes them ideal candidates for pest-specific biopesticides with no or minimal harmful effects on closely related nontarget organisms.

In this study, we demonstrated that lectins from fungal fruiting bodies exert toxic effects on CPB larvae, including increased mortality and decreased food intake and growth. Some of these lectins were previously investigated in the parasitic nematode *Haemonchus contortus*. Dose-dependent toxicity of CGL2, AAL, and MOA was reported: larval development was strongly inhibited at lectin concentrations as low as 1 μg/mL and almost completely (>98%) prevented at 5 μg/mL.^22^ MOA was the only lectin that caused larval death, whereas the other toxic lectins exerted a larvistatic effect. In our study, MOA had the most pronounced toxic effect on CPB larvae, increasing mortality and decreasing weight. Moreover, nematocidal effects of MOA have been demonstrated in *C. elegans* larvae.^21^ As most nematodes are much smaller than CPB larvae, the toxicity caused by the same MOA concentrations is not directly comparable. Thus, we compared MOA with the bioinsecticide Novodor FC (which is based on the entomopathogenic bacterium *B. thuringiensis* subsp. *tenebrionis*). Novodor FC had a larval mortality rate of 78%, i.e., approximately 20% lower than that of MOA.

MOA expression in transgenic plants can significantly increase resistance to the herbivorous insect *Plutella xylostella* (Lepidoptera), one of the main pests of brassicas.^19^ The efficacy of pure lectin extracts cannot be directly equated to that of lectins in plants. Nevertheless, the survival rate of *P. xylostella* larvae exposed to MOA-expressing plants was decreased by 15–24% compared to the control after 7 days, depending on larval age. In our study, the mortality rate of CPB larvae was almost 100% after only 5 days of exposure. In another study in which CPB larvae were fed with extracts of the basidiomycete *C. nebularis*, only CNL lectin showed antifeeding effects.^8^ A negative effect on larval weight gain, which did not lead to increased larval mortality, was demonstrated when leaves were immersed in 2.2 mg/mL (but not 1.1 mg/mL) CNL. In our study, 1 mg/mL MOA decreased the survival rate by approximately 75% compared to the control group. The amount of MOA added to the ingested food was estimated to be 0.020% (w/w, for 1 mg/mL MOA solution), which is comparable to the 2.2-fold higher concentration of CNL (0.018%, for 2.2 mg/mL CNL solution) tested in a previous study.^8^

In addition to lectins, higher fungi contain a high proportion of protease inhibitors, which represent another important group of phytoprotective proteins. Fungal cysteine protease inhibitors from *Macrolepiota procera* (macrocypins)^32^ and *C. nebularis* (clitocypin)^31^ were tested for their potential to control CPB larvae. Larvae were fed with inhibitor-coated leaves or on transgenic plants expressing these fungal protease inhibitors. Larval growth and weight gain were significantly decreased, whereas survival rates did not differ significantly between control and test groups. Only a delay in development was observed.^31^

Among nonfungal lectins, the plant lectin Gleheda, isolated from ground ivy (*G. hederacea*), was the first to be investigated for its insecticidal activity against CPB larvae.^30^ Its antifeeding activity was demonstrated by dipping leaves in a 20 mg/mL lectin solution, which resulted in inhibited food intake, decreased weight gain, and complete mortality of larvae after 7 days. In our study, similar negative effects of MOA were observed at a 20-fold lower concentration (1 mg/mL).

The differences in the toxicities of various lectins depend on the specificity of carbohydrate binding. As the MOA mutants lacking carbohydrate binding showed no or significantly decreased toxicity, we conclude that this binding activity is essential for MOA toxicity. These results are in complete agreement with the results of a previous study.^21^ In particular, the lack of toxicity of MOA Q46AW138A and MOA C215A indicates that, similar to nematotoxicity, the entomotoxicity of MOA depends on both its carbohydrate-binding and proteolytic activity. This is also supported by the decreased toxicity of MOA ΔC, a mutant containing only the carbohydrate-binding domain. This is possibly due to either loss of dimerization or loss of proteolytic activity. Such a loss of proteolytic activity also resulted in lower toxicity against the mammalian cell line NIH/3T3,^61^ confirming the importance of the protease domain for the cytotoxicity of MOA. The proteolytic activity of MOA may also explain why MOA exerts a stronger effect than that of the Bt bioinsecticide, as such activity can target multiple intracellular targets, unlike the pore-forming activity of Cry toxins.^62^ No inhibition of toxicity was observed when the Me-α-Gal monosaccharide was used as an inhibitory sugar to prevent MOA binding to the target carbohydrate, which should mimic the effects of MOA Q46AW138A. This is likely due to the low affinity for binding to Me-α-Gal compared to endogenous target glycans. To this end, the disaccharide Gal-α1,3-Gal or trisaccharide Gal-α1,3-Gal-β1,4-GlcNAc should probably be used to observe inhibition of MOA binding. Conversely, galactose decreased the antiproliferative effects of MOA against mammalian cancer cells.^63^

Interestingly, the target glycoconjugate of MOA in CPB larvae is not a glycolipid, as has been shown for *C. elegans*,^21^ but a glycoprotein. Aminopeptidase, a 112 kDa glycoprotein localized at the plasma membrane, is likely the target glycoconjugate that enables MOA internalization in CPB larvae. Similarly, aminopeptidase N was shown to be the target glycoconjugate for *M. procera* lectin (MpL), which was internalized by endocytosis after interaction with aminopeptidase N on human cancer cells.^64^ The internalization of aminopeptidase N is induced by the binding of antibodies^65^ and various viruses (e.g., coronaviruses^66–68^ and cytomegalovirus^69^) in mammalian cells. In insects, aminopeptidase has been identified as one of the receptors for *B. thuringiensis* Cry toxins in Lepidoptera,^70^ Coleoptera,^71^ and Diptera.^72^

Integrins are involved in the binding of MpL,^64^ and MOA induces the internalization and degradation of integrins in mammalian Madin-Darby canine kidney strain II (MDCKII) cells.^73^ However, no direct interaction of integrins with MOA has been demonstrated in CPB larvae. Furthermore, MOA is mainly internalized into MDCKII cells by clathrin-mediated endocytosis and accumulates in late endosomal compartments, leading to impaired cell adhesion signaling, integrin degradation, and thereby decreased cell viability.^73^ MOA was shown to bind to clathrin in protein extracts from CPB larvae, indicating a possible target of its proteolytic activity. Other protein targets include stress response proteins (e.g., heat shock proteins 60 kDa, 70a, and 83 and E1-ubiquitin-activating enzyme) and proteins involved in metabolism either intracellularly or extracellularly (e.g., multifunctional fusion protein, family 48 glycosidase, protease cathepsin B, and carboxylic ester hydrolase). Intracellular proteins are likely targets of the proteolytic activity of MOA, whereas extracellularly these proteins (e.g., yellow-x2, cathepsin B, carboxylic ester hydrolase, and family 48 glycosidase) may act as decoys and prevent MOA from interacting with epithelial cells.

Microscopic characterization of anatomical, histological, and ultrastructural modifications following agent treatment in intact organisms provides valuable information on the mode of action of potential entomotoxins in a physiologically complex environment. The insect midgut epithelium, at the interface between the external and internal environments, is essential for digestion, nutrient absorption, electrolyte homeostasis, and protection against pathogens.^74^ Impairment of gut structure and thus function is a potential basis for innovative pest control measures, and deleterious effects of Bt toxins on the midgut epithelium have already been demonstrated.^75,76^ Lectins have also been reported to exert various deleterious effects on the structure of the insect midgut,^13^ damaging microvilli and midgut structure already at the histological level.^77–79^

We observed a severe disruption of midgut epithelial organization in MOA-exposed CPB larvae. Midgut cells lost their polarity, resulting in a loss of the microvillous brush border and basal labyrinth as well as aberrant positioning of the septate junctions and rounding of the cells. These damaged cells detached from the midgut epithelium and accumulated in the midgut lumen, where they disintegrated. Basally, beneath the detached midgut cells, we observed the accumulation of numerous small cells that roughly resembled stem cells of the midgut epithelium of the control larvae. Similar delamination of damaged enterocyte-like cells and activation of stem cell division in the midgut epithelium has been observed in western corn rootworm larvae exposed to Bt toxins^80^ and in *Drosophila* exposed to sodium dextran sulfate^81^ and after bacterial infection.^82,83^ This appears to represent a general program of epithelial renewal triggered in response to midgut cell damage.^84,85^ However, this response is apparently insufficient to keep pace with the extensive damage caused by MOA, resulting in larval mortality.

The detachment of midgut epithelial cells could also be a direct effect of MOA. MOA can cause detachment of mouse glomerular microvascular endothelial cells,^86^ canine MDCKII cells,^73^ and human SW1573 lung cancer cells.^63^ According to Juillot et al. (2016),^73^ MOA causes internalization and degradation of β1-integrin and disruption of focal adhesion signaling, leading to cytoskeletal restructuring, cell detachment, and cell death. Our analysis identified actin as a potential target of MOA. Actin stress fibers in focal adhesions of vertebrate cells^87,88^ and actin fiber arrays in junctions of insect cells^89^ are important in integrin-mediated cell adhesion to the extracellular matrix. We observed that in MOA-treated CPB larvae, the basal lamina detached from the basal plasma membrane of midgut cells, and hemidesmosome-like junctions were absent, indicating impaired integrin-dependent cell adhesion.

The disruptive effects of MOA were limited to the midgut and were not observed in the hindgut. The hindgut, in contrast to the midgut, is lined by a chitinous cuticle on the luminal side,^74,90^ which acts as a molecular sieve that restricts the passage of large molecules, including toxic waste products and xenobiotics.^91^

Screening tests on adult and larval honeybees assessed the acute oral toxicity of various lectins and showed no adverse effects. There were no discernible variations in adult bee survival or food intake, indicating that the tested lectins do not cause acute toxicity in adult honeybees at the tested concentrations. Additionally, the LC_50_ value of MOA, calculated in accordance with OECD guidelines,^54^ exceeded 100 μg a.i./bee, suggesting that MOA is not acutely toxic to adult honeybees. Both glycan binding and proteolytic activity are required for full MOA toxicity in target organisms, which may explain the different effects between CPB and honeybee. This selective toxicity may result from the absence or decreased availability of specific glycan targets. Glycosylation profiles, specifically N-glycomes, differ between CPB and honeybees. N-glycans in the peritrophic membrane of CPB larvae mainly consist of paucimannoses, monofucosylated paucimannoses, and high mannoses.^92^ Conversely, N-glycans in honeybee larvae consist of oligomannosidic and paucimannosidic structures, together with a range of modified glycans carrying glucuronic acid, sulfate, or phosphoethanolamine groups.^93^ In addition, differences in the gut environment, including pH, ion composition, and the presence of proteolytic enzymes or neutralizing agents,^94^ can affect the insecticidal activity of ingested lectins. This highlights the importance of host-specific glycan expression and gut physiology in determining susceptibility to lectin-based toxins, while also indicating their high potential for selectivity.

The limit test method supported the designation of MOA as a low-toxicity substance in honeybee, as no appreciable mortality was observed within the tested range (1–3 mg/mL). These results validate the sensitivity and reliability of this approach in identifying potential harmful effects. Toxicity assays vary depending on the distinct feeding behaviors of different insects. When interpreting toxicity results, it is important to consider these differences and the variation in exposure time, as both factors can influence the observed toxicity outcome. In addition, ontogenetic differences must be considered when assessing the ecological risks of lectins. For example, bee larvae experience significant developmental effects, whereas adult bees do not. The effects on larval weight are consistent with previous research showing that lectins can interfere with midgut glycoproteins, nutrient absorption, and hormone regulation, thereby disrupting insect growth and metabolism. Although the specific mechanisms are still unknown, previous research indicates that lectins may affect development by binding to gut epithelial cells.^95^ To clarify the mechanisms underlying these effects and ascertain whether prolonged exposure is harmful to honeybees, more research is required, including histopathological and enzymatic analyses.

In conclusion, the fungal lectins MOA, AAL, CCL2, and CCP1 impair the feeding rate of CPB larvae. MOA exhibited the strongest insecticidal activity, which requires both glycan binding and proteolytic activity. In contrast to glycolipids as molecular targets in nematodes, the glycoprotein aminopeptidase was identified as a putative molecular target of MOA in the midgut of CPB larvae. MOA caused complete disorganization of the midgut epithelium, which was lethal to CPB larvae. Given the incredible adaptability of CPB to pesticides/xenobiotics,^28^ it is important to understand the mechanism of action of potential biopesticides, as this can inform their further development, integration with other compatible biopesticides, and potential applications. In addition, when assessing potential biopesticides, it is also important to include (agro)­ecologically important nontarget organisms, such as honeybees, already in the early research stages. In the current study, MOA proved to be insecticidal to CPB larvae with no toxicity against adult honeybees, confirming higher fungi as a perspective source of selective insecticidal proteins.

## Supplementary Material



## Data Availability

The data sets generated and/or analyzed during the current study are available from the corresponding author on reasonable request.

## Notes

Additional supporting experimental details are provided in nine supplementary figures (SDS–PAGE analyses of *Marasmius oreades* agglutinin (MOA) affinity chromatography eluates and purified recombinant fungal proteins; representative images and results of feeding bioassays with Colorado potato beetle (CPB) larvae, including excised potato leaf disc assays, mortality, and LC_5__0_ determination; and surface plasmon resonance analyses of MOA binding to CPB larval gut lipid extracts and large unilamellar vesicles; sequences and mass spectrometry-identified peptides of MOA molecular target proteins; feeding trials and representative images of *Apis mellifera* larvae) and three supplementary tables (MOA mutants and reported functionality; identification of proteins bound to MOA in CPB larval gut extracts by peptide mass fingerprinting from two independent experiments) (PDF)

## §

National Institute of Biology, 1000 Ljubljana, Slovenia

## Abbreviations

AAL: *Aleuria aurantia* lectin
CCL2: *Coprinopsis cinerea* lectin 2
CCP1: Cocaprin 1
CGL2: *Coprinopsis cinerea* galectin 2
CGL3: *Coprinopsis cinerea* galectin 3
CPB: Colorado potato beetle
MOA: *Marasmius oreades* agglutinin
