# Supplementary material for: The Entomotoxic Fungal Lectin Marasmius oreades Agglutinin Disrupts the Midgut Epithelium of Colorado Potato Beetle Larvae
Source: J Agric Food Chem. 2026 Feb 17;74(8):6890–904. doi: 10.1021/acs.jafc.5c16986 (PMC12964528; doi:10.1021/acs.jafc.5c16986)
Supplement: Supplementary file 1 [file jf5c16986_si_001.pdf]

The Entomotoxic Fungal Lectin *Marasmius oreades* Agglutinin Disrupts the Midgut Epithelium of Colorado Potato Beetle Larvae

Primož Žigon, Urban Bogataj, Sergej Praček, Tjaša Peternel, Polona Mrak, Maja Ivana Smodiš Škerl, Márta Ladányi, Katarina Karničar, Marko Fonović, Anastasija Panevska, Matej Skočaj, Dušan Turk, Nada Žnidaršič, Jaka Razinger* jaka.razinger@kis.si, Jerica Sabotič* jerica.sabotic@ijs.si

* corresponding authors; shared lead authorship

Affiliations

Agricultural Institute of Slovenia, Plant Protection Department, Ljubljana, Slovenia
Primož Žigon & Jaka Razinger

University of Ljubljana, Biotechnical Faculty, Department of Biology, Ljubljana, Slovenia
Urban Bogataj, Polona Mrak, Anastasija Panevska, Matej Skočaj, Nada Žnidaršič

Jožef Stefan Institute, Department of Biotechnology, Ljubljana, Slovenia
Sergej Praček, Tjaša Peternel, Jerica Sabotič

National Institute of Biology, Ljubljana, Slovenia
Sergej Praček

Agricultural Institute of Slovenia, Animal Production Department, Ljubljana, Slovenia
Maja Ivana Smodiš Škerl

Hungarian University of Agriculture and Life Sciences (MATE), Department of Applied Statistics, Institute of Mathematics and Basic Science, Budapest, Hungary
Márta Ladányi

Jožef Stefan Institute, Department of Biochemistry and Molecular and Structural Biology, Ljubljana, Slovenia
Katarina Karničar, Dušan Turk, Marko Fonović

Centre of Excellence for Integrated Approaches in Chemistry and Biology of Proteins, Ljubljana, Slovenia
Katarina Karničar & Dušan Turk

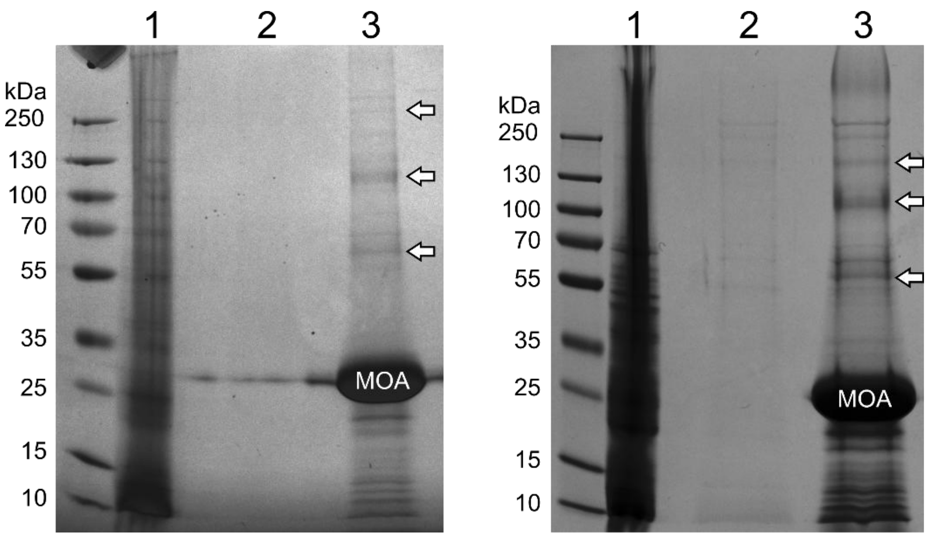

Figure S1 SDS-PAGE analysis of two repeated *Marasmius oreades* agglutinin (MOA)-affinity chromatography eluates visualized by Coomassie blue staining. Lane 1: protein extract of Colorado potato beetle larval guts that was loaded to affinity chromatography. Lane 2: negative control, where only Sepharose was used for affinity pull-down. Lane 3: MOA-Sepharose-affinity pull-down; bands excised for mass spectrometry are indicated by white arrows. MOA that eluted from Sepharose with boiling in sample buffer is also indicated. The PageRuler Plus Prestained Protein Ladder 10–250 kDa (Fisher Scientific) was used.

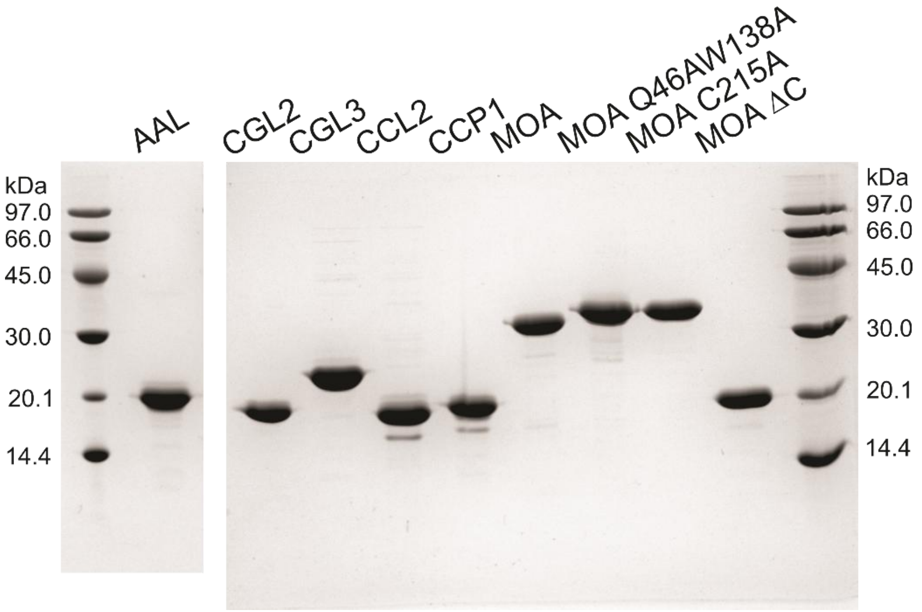

Figure S2 Purified recombinant fungal proteins (4 µg per lane) were analysed by SDS-PAGE (15%) and visualized by Coomassie staining. Abbreviations of protein names are defined in Table 1. Additional bands are observed for CCL2 and CCP1, corresponding to each protein. This is attributed to variations in electrophoretic mobility rather than sample impurity, as previously noted for fungal β-trefoil proteins and confirmed by mass spectrometric analysis of a representative sample. The Amersham Low Molecular Weight Calibration Kit for SDS Electrophoresis (GE Healthcare) was used.

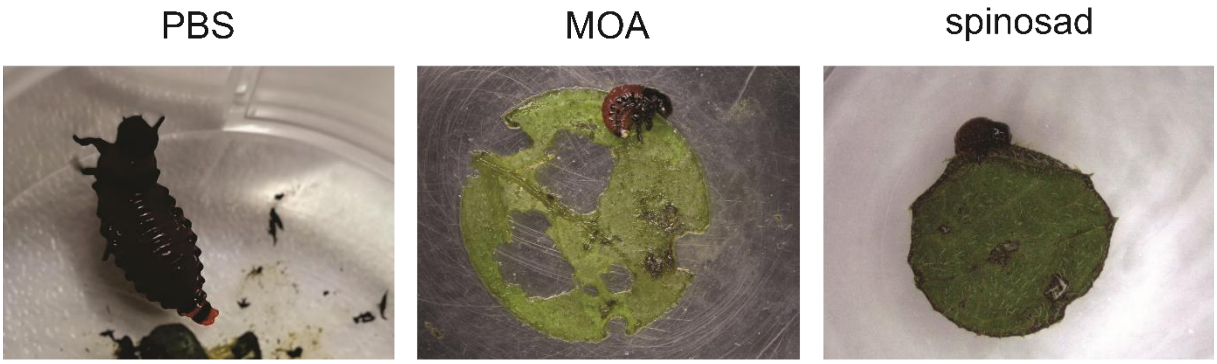

Figure S3 Representative images of Colorado potato beetle larvae on the third day of feeding bioassays with treated, excised potato leaf discs soaked in solutions of phosphate-buffered saline (PBS) as a negative control, *Marasmius oreades* agglutinin (MOA) at 1 mg/mL, and spinosad insecticide as a positive control.

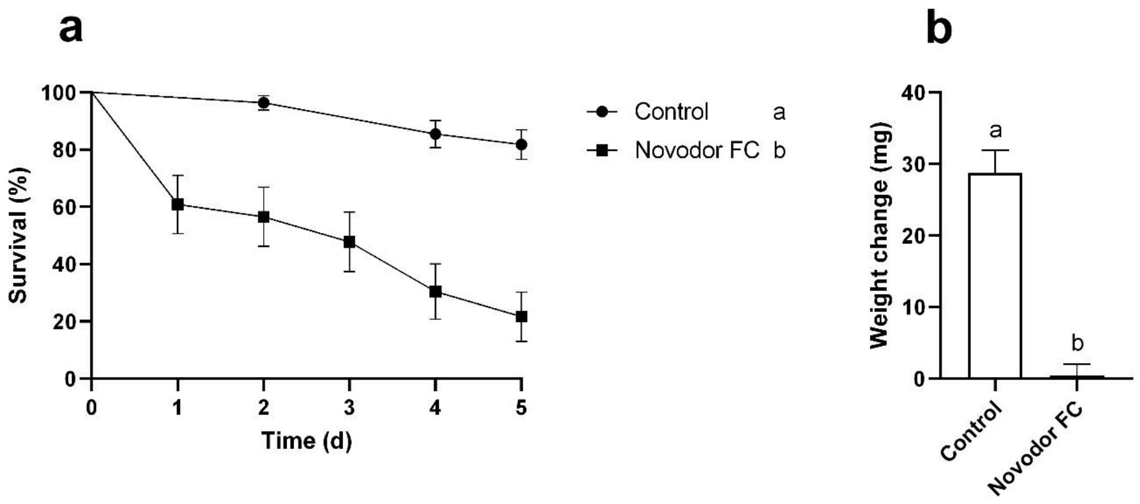

Figure S4 Feeding trials with Colorado potato beetle (CPB) larvae on excised potato leaf discs treated with Novodor FC and a negative control (phosphate-buffered saline). The trials lasted 5 days. (a) Survival curves and (b) weight changes of CPB larvae. Different lowercase letters indicate significant differences between treatments ($P < 0.05$; a: Mantel–Cox test, b: unpaired t-test).

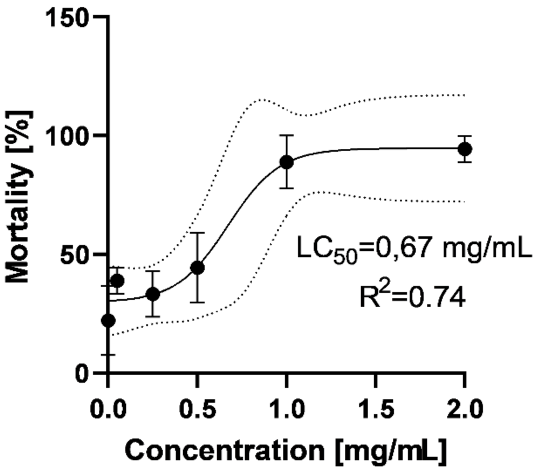

Figure S5 Mortality (%) and lethal concentrations (LC_{50}) (mg mL^{-1}) of *Marasmius oreades* agglutinin against Colorado potato beetle larvae (L2, after 48 h). The dotted lines denote the 95% confidence interval of the best-fit line.

Table S1 *Marasmius oreades* agglutinin (MOA) mutants were selected based on their previously described functionality (Wohschlager et al., 2011).

MOA variant	**Glycan-binding**	**Dimerization**	**Proteolytic activity**
**MOA**	✓	✓	✓
**MOA Q46AW138A**	✗	✓	✓
**MOA C215A**	✓	✓	✗
**MOA ΔC**	✓	✗	✗

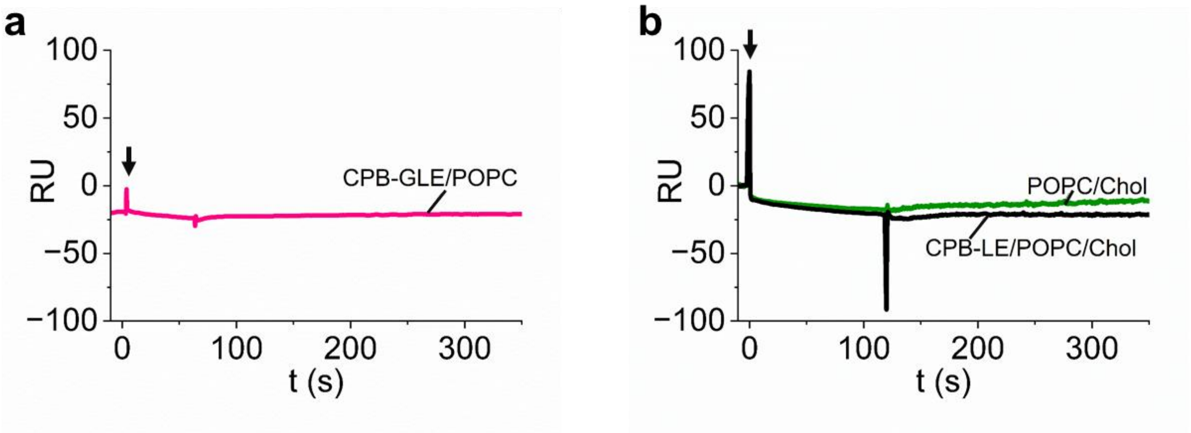

Figure S6 Surface plasmon resonance sensorgrams showing *Marasmius oreades* agglutinin (MOA) binding kinetics to Colorado potato beetle (CPB) larval gut lipid extract and large unilamellar vesicles (LUVs) of whole-larvae lipid extract. (**a**) LUVs composed of CPB larval gut polar lipid extract (CPB-GLE) and 1-palmitoyl-2-oleoyl-sn-glycero-3-phosphocholine (POPC) (1:1 molar ratio) were immobilized on an L1 sensor chip (7,000–8,000 response units (RU)). MOA (10 µM) was injected at a flow rate of 10 µL/min for 60 s (association) followed by a 290 s dissociation phase. (**b**) LUVs composed of whole-larvae polar lipid extract (CPB-LE), POPC, and cholesterol (1:1:1 molar ratio) were immobilized on an L1 sensor chip (7,000–8,000 RU). MOA (10 µM) was injected at a flow rate of 10 µL/min for 120 s (association) followed by a 230 s dissociation phase. POPC:cholesterol (1:1 molar ratio) vesicles served as a negative control. Background-subtracted sensorgrams averaged from triplicate measurements are shown, with standard errors below 5%.

Table S2 Proteins bound to *Marasmius oreades* agglutinin (MOA) in the gut extract of Colorado potato beetle (CPB) larvae identified by peptide mass fingerprinting in the first experiment. Proteins detected in both replicates are shown in bold.

Protein ID and name	Unique peptides	Molecular/biological function	Biological process	Subcellular location
**U6BQX5_LEPDE Heat shock protein 70a**	**21**	**chaperone**	**stress response**	**cytoplasm**
**E7CIZ1_LEPDE Glycoside hydrolase family protein 48**	**17**	**glycosidase**	**carbohydrate metabolism, polysaccharide degradation**	**extracellular**
E7CIZ0_LEPDE Glycoside hydrolase family protein 48	16	glycosidase	carbohydrate metabolism, polysaccharide degradation	extracellular
**A0A2D1QUF3_LEPDE Clathrin heavy chain**	**12**	**structural molecule**	**intracellular protein transport, vesicle organization**	**membrane, cytoplasmic vesicle membrane**
**V5QQR2_LEPDE 60 kDa heat shock protein**	**10**	**chaperone**	**stress response**	**mitochondrial matrix**
**A0A0A7ENR4_LEPDE Carboxylic ester hydrolase**	**8**	**carboxylic ester hydrolase**	**lipid metabolism**	**cytoplasm, extracellular**
**A0A0E3ISE4_LEPDE Actin**	**7**	**cytoskeleton molecule**	**actin filament organization**	**cytoplasm**
**D9J2F5_LEPDE Aminopeptidase**	**7**	**metalloaminopeptidase**	**proteolysis**	**cell membrane (gpi-anchor)**
**Q3I414_LEPDE Cytochrome P450**	**6**	**oxidoreductase**	**p450-containing electron transport chain**	**endoplasmic reticulum membrane**
**V5QPM9_LEPDE Heat shock protein 83**	**5**	**unfolded protein binding, chaperone**	**stress response**	**cytoplasm**
A0A0A7ENP2_LEPDE Carboxylic ester hydrolase (Fragment)	4	carboxylic ester hydrolase	lipid metabolism	cytoplasm, extracellular
A0A0A7ENS3_LEPDE Carboxylic ester hydrolase	4	carboxylic ester hydrolase	lipid metabolism	cytoplasm, extracellular
V9PB47_LEPDE Sulfhydryl oxidase (Fragment)	4	oxidoreductase	protein folding	extracellular, Golgi membrane
**A0A290GAZ2_LEPDE Yellow-x2**	**3**	**major royal jelly protein family**	**larval or pupal development**	**extracellular**
A0A8B0L9I3_LEPDE Calnexin	3	chaperone	protein folding	endoplasmic reticulum membrane
**V9PBG4_LEPDE Cathepsin B**	**3**	**cysteine-type peptidase activity**	**proteolysis**	**lysosome, extracellular**
**A0A2D1QUE3_LEPDE E1 ubiquitin-activating enzyme**	**2**	**ubiquitin-activating enzyme**	**DNA damage response, protein catabolic process**	**cytoplasm, nucleus**
**G9FQ75_LEPDE Multifunctional fusion protein**	**2**	**oxidoreductase**	**proline metabolism**	**mitochondrial matrix**

Table S3 Proteins bound to *Marasmius oreades* agglutinin (MOA) in the gut extract of Colorado potato beetle (CPB) larvae identified by peptide mass fingerprinting in the second experiment. Proteins detected in both replicates are shown in bold.

Protein ID and name	Unique peptides	Molecular/biological function	Biological process	Subcellular location
**Q3I414_LEPDE Cytochrome P450**	**19**	**oxidoreductase**	**p450-containing electron transport chain**	**endoplasmic reticulum membrane**
A0A1W5YLK4_LEPDE Pyruvate kinase	11	kinase activity	glycolysis	cytoplasm
**A0A0E3ISE4_LEPDE Actin**	**10**	**cytoskeleton molecule**	**actin filament organization**	**cytoplasm**
**U6BQX5_LEPDE Heat shock protein 70a**	**10**	**chaperone**	**stress response**	**cytoplasm**
**V9PBG4_LEPDE Cathepsin B**	**9**	**cysteine-type peptidase activity**	**proteolysis**	**lysosome, extracellular**
A0A2D1QUC6_LEPDE ATP synthase subunit beta	8	translocase	ion transport	mitochondrion
**A0A2D1QUF3_LEPDE Clathrin heavy chain**	**8**	**structural molecule**	**intracellular protein transport, vesicle organization**	**membrane, cytoplasmic vesicle membrane**
**E7CIZ1_LEPDE Glycoside hydrolase family protein 48**	**8**	**glycosidase**	**carbohydrate metabolism, polysaccharide degradation**	**extracellular**
Q6UZ77_LEPDE Bifunctional purine biosynthesis protein ATIC	8	hydrolase, transferase	purine biosynthesis	cytosol
**V5QQR2_LEPDE 60 kDa heat shock protein**	**8**	**chaperone**	**stress response**	**mitochondrial matrix**
**D9J2F5_LEPDE Aminopeptidase**	**7**	**metalloaminopeptidase**	**proteolysis**	**cell membrane (gpi-anchor)**
O44707_LEPDE Juvenile hormone esterase-related protein	6	juvenile hormone esterase	juvenile hormone metabolism	extracellular
V9PBD6_LEPDE Dipeptidyl peptidase	6	peptidase activity	proteolysis	plasma membrane
Q25271_LEPDE Diapause protein 1 (Fragment)	5	storage protein	metabolic and diapause regulation	extracellular
A0A0F6P322_LEPDE Cytochrome P450	4	oxidoreductase	p450-containing electron transport chain	endoplasmic reticulum membrane
A0A1W5YLJ2_LEPDE Glucose-6-phosphate isomerase	4	monosaccharide binding	gluconeogenesis, glycolytic process	cytosol
**G9FQ75_LEPDE Multifunctional fusion protein**	**4**	**oxidoreductase**	**proline metabolism**	**mitochondrial matrix**
O44123_LEPDE Putative juvenile hormone esterase	4	juvenile hormone esterase	juvenile hormone metabolism	extracellular
Q6QRP7_LEPDE Digestive cysteine proteinase intestine	4	cysteine-type peptidase activity	proteolysis	extracellular
**A0A0A7ENR4_LEPDE Carboxylic ester hydrolase**	**3**	**carboxylic ester hydrolase activity**	**lipid metabolism, xenobiotic metabolism**	**endoplasmic reticulum**

Protein ID and name	Unique peptides	Molecular/biological function	Biological process	Subcellular location
A0A1P8PEX0_LEPDE Putative glutathione S-transferase sigma class member 5b	3	glutathione transferase activity	glutathione metabolic process	cytosol
**A0A290GAZ2_LEPDE Yellow-x2**	**3**	**major royal jelly protein family**	**larval or pupal development**	**extracellular**
**A0A2D1QUE3_LEPDE E1 ubiquitin-activating enzyme**	**3**	**ubiquitin-activating enzyme**	**DNA damage response, protein catabolic process**	**cytoplasm, nucleus**
A0A2Z4N548_LEPDE Chitin deacetylase 6	3	hydrolase activity	carbohydrate metabolism	extracellular
A0A8B0L7G8_LEPDE Calreticulin	3	carbohydrate binding, unfolded protein binding	protein folding, ERAD pathway	endoplasmic reticulum lumen and membrane
**V5QPM9_LEPDE Heat shock protein 83**	**3**	**unfolded protein binding, chaperone**	**stress response**	**cytoplasm**
A0A0A7ENL4_LEPDE Carboxylic ester hydrolase (Fragment)	2	carboxylic ester hydrolase activity	lipid metabolism, xenobiotic metabolism	endoplasmic reticulum
A0A0A7EP48_LEPDE Putative alpha-esterase	2	serine esterase	lipid metabolism, xenobiotic metabolism	extracellular
A0A0F6TN63_LEPDE Alpha-amylase (Fragment)	2	alpha-amylase activity	carbohydrate metabolism	extracellular
A0A1D8I2M3_LEPDE Trehalose-6-phosphate synthase	2	trehalose-phosphatase activity	trehalose biosynthesis, trehalose metabolism in response to stress	cytosol
A0A1W5YLL3_LEPDE Isocitrate dehydrogenase [NADP]	2	isocitrate dehydrogenase (NADP+) activity	glyoxylate cycle, isocitrate metabolism, NADP metabolism, TCA cycle	cytosol
A0A1W5YLL4_LEPDE Glucose-6-phosphate 1-dehydrogenase	2	glucose-6-phosphate dehydrogenase activity, NADP binding	glucose metabolism	cytosol
A2I7Q3_LEPDE Digestive cysteine protease intestine (Fragment)	2	peptidase activity	proteolysis	extracellular
V5K4Z6_LEPDE Cytochrome P450 6bq16 (Fragment)	2	oxidoreductase	p450-containing electron transport chain	endoplasmic reticulum membrane
V5K4Z9_LEPDE Cytochrome P450 6ed1 (Fragment)	2	oxidoreductase	p450-containing electron transport chain	endoplasmic reticulum membrane

Figure S7 Sequences of proteins identified as molecular targets of *Marasmius oreades* agglutinin (MOA) (Table 2) and the peptides detected with mass spectrometry. Peptides identified in the first, second, and both experiments are highlighted in yellow, blue, and green, respectively. Similar peptides with small differences in length in each experiment are underlined. Putative glycosylation sites with high prediction scores are indicated in red, as determined using the online tools NetNGlyc 1.0 (https://services.healthtech.dtu.dk/services/NetNGlyc-1.0/) (Gupta and Brunak, 2002) or NetOGlyc 4.0 (https://services.healthtech.dtu.dk/services/NetOGlyc-4.0/) (Steentoft et al., 2013). Sites located within the identified peptides are unlikely to be glycosylated in our samples.

>D9J2F5_LEPDE **Aminopeptidase APN1**

MASSTIILIILFGYTPCVFSQINNTDLEYRLPNIYIPTQYTLNLQLPESAFMAEDSEYSGSVLIVFNVSETVSQVKIHANP
VLQITGVNLVGIGETNFTVEDATEILTIHSSTALQAGNTYLINITFNGLLRTDDMTGLYKSSYIDNTAGVTKYLITTQFQ
PTSARKAFPCFDEPSFKASFTVSVDCPSNFTVHSNAAIRSSTITNGTKRTVFESTPPMSTYLLAILVSEFSCTEKVILANI
TSAVCSRNESSELRSWAEEVTPKLLSFFNNYTGLNYNDYLSHLYQVAIPDFANGAMENWGLVSYREEFLLWDPLESS
NLFKQLVATIISHELAHKWFGNLVTLEWWSELFLNEGLATYFEYFATHEVLPEFQLDKQFVIDVLQSVMRDDSENISP
LQSNVSSPAEVMSKFSRISYFKGGSIIRMVEHIIGSSDFRAGIQDYLTTNALGNVISEQLWTHLQANMTNSNALPTTL
NVIMKDWIQQPGFPLITATLQNQGVLVLTQSTFSISDNSTSQWFIPISYTKSSDLMKFNSTSPKVWLTPNGTANITL
MQGDSWIILNNQVTGYFRVNYDNELWSRIGTALKSENFSDIPEIHRAQIVDDLFNLARIQKTNYSQVFDLIEFLANDT
SYFSWKVAFDGFNFLLERVGSDSVLGQQISKHLLRLMTNLYDSTSFDRLNDTYHIDILKQGLTLSWACSLGNSQCIEA
SRNRYSSYKMNGTRPDKNLRNIVYCNGLRDDTSSSNWDFLWNKYLNTTIATEKITILSSLGCTKNVTLLEGYLNKTLR
SDSGIRPKDRFEVFSSVLNGNTIGIDITLNFLVTNPLQVVQKYPNFNSISRIISNIANKITTQNQVDQLKQFIWSGELGN
EHLLSANNSLATAEANLLWMNYFREHLHNYYRTPNATNGTTTSIPTSDTTPTAITNGTIPTTITNNTTITTITNGTTPA
SITNGTTPTTITNGTTSATSTITPSPIPAGSESIVKVNRLSIVSMILCYIMII

>A0A2D1QUF3_LEPDE **Clathrin heavy chain**

MTQQLLPIKFQEHLQLTNVGINAANITFATLTMESDKFICVREKVGEISQVVIIDMADTSNPIRRPITAESAIMNPASK
VIALKGIGKAGVETQKTLQIFNIEMKSKMKAHTMSEDVIFWKWISPNTLALVTETSVYHWSMEGDSTPQKMFDRH
SSLNGCQIINYRTDPKQNWLLLVGISAQQSRVVGAMQLYSVERKCSQPIEGHAASFATFKMEGNPEPSTLFCFAVR
TLQGGKLHIIEVGQSPAGNQPFPKKTVDVFFPPEAQNDFPVAMQVSAKYDVIYLITKYGYIHMYDIESATCIYMNRIS
SETIFVTAPHEVSGGIIGVNRRGQVLSVSVDEESIIRYVNTILHNADLALRMAIRSNLAGAEELFVSKFQMLFQNGQY
AEAAKVAANAPKGILRTPATIQMFQQVPTQAGQNSPLLQYFGILLDQGKLNRYESLELCKPVLLQGRKQLLEKWLKE
DKLECSEELGDLVKQADPTLALSVYLRANVPAKVIQSFAETGQFQKIVLYAKKVSYTPDYIFLLRQVMRTNPDQGAAF
AGMLVADEEPLADINQIVDIFMEQNMVQQCTAFLLDALKNNRPTEGHLQTRLLEMNLMSAPQVADAILGNNMF
THYDRPHIAQLCEKAGLLQRALEHYTDLYDIKRAVVHTHLLPADWLVNFFGTLSVEDSLECLKAMLTANIRQNLQIC
VQIATKYHEQLTTKALIDLFESFKSYEGLFYFLGSIVNFSQDQEVHFKYIQAACKTGQIKEVERICRESNCYNPDRVKNF
LKEAKLTDQLPLIIVCDRFDFVHDLVLYLYRNSLQKYIEIYVQKVNPSRLPVVVGGLLDVDCSEDIIKNLILVVRGQFSTD
ELVEEVEKRNRLKLLLPWLESRVHEGCVEPATHNALAKIYIDSNNNAERFLKENQWYDSRVVGRYCEKRDPHLACV
AYERGQCDRELIAVCNENSLFKSEARYLVRRRDGELWAEVLQESNPYRRQLIDQVVQTALSETQDPEDISVTVKAFM
TADLPNELIELLEKIVLDTSVFSEHRNLQNLLILTAIKADATRVMDYINRLDNYDAPDIANIAINNHLYEEAFAIFRKFDV
NTSAIQVLIEQVNNLDRAYEFAERCNEPAVWSQLAKAQLNQGLVKEAIDSYIKADDPSAYMAVVETASQNNSWED
LVRYLQMARKKARESYIESELIYSYAKTGRLADLEEFISGPNHADIQKIGDRCFDDKMYDAAKLLYNNVSNFARLAITL
VHLKEFQGAVDSARKANSTRTWKEVCFACVDAEEFRLAQMCGMHIVVHADELQDLINYYQDRGYFEELIGLLEAAL
GLERAHMGMFTELAILYSKYKPAKMREHLELFWSRVNIPKVLRAAEQAHLWAELVFLYDKYEEYDNAVLAMMAHP
TEAWREGHFKDIITKVANIELYYKAIQFYLDYKPLLLNDLLLVLAPRMDHTRAVSFFSKTGHLQLVKSYLRSVQNLNNK

AINEALNSLLIEEEDFQGLRTSIDAFDNFDNIGFGQKLEKHELTEFRRIAAYLYKGNNRWKQSVELCKKDRLFRDAME
YTAESRNQELAEELLAWFLERKAYDCFSACLYQCYDLLRPDVILELAWKHKIMDFAMPYLIQVTRELTTKVEKLEQSE
AERRNEAAEETNKPMMIPEPQLMLTAGPGMGIPPQQYVPPQAYPQPGYAPQMPYQGYPGM

>Q3I414_LEPDE **Cytochrome P450**

MVFSTDNLLLDLFGVLVALSAVLFVYIKRSFQYWDRKGVPYLPPNIPWGNLQPPHSRDIPEGDDVANIYYKAKAKG
WKYIGIYVMTGSVFLPVDLELIKHITTKDFQHFVDRGTYVNEKDEPITAHLFSIGGKKWRNLRTKFTPTFTSGKMRQ
MFETIANCGHILEKYIEHEVDHHEPLDIKNVLACYTTDIIGSCAFGLDCNSFKEPNSPFTQFGQRVFRTDGIRNLKITF
MGAFPNLSKMLRMRLTEKEVEDFYTKVVEDTVRYREKEGVTRPDFLQMLIDIKNKTNEHTGDGTSLTMDEIVAQSF
VFFIAGFETSSTTMTFALYQLATHPEIQEKVRSEINSVLEKHNNQITYDALNELKYMGKVIDETLRMYPALPVVTRRC
VEDYRIPDSDVIIEKGIEVFIPIKAIHYDPEYYENPEVFDPERFNEENIQGRHPYAHIPFGEGPRICIGLRFGVMQSKVGL
VSILKNFRVTLSSKTKLPLKIDVNSFIPTTEGGMWLNLERIGK

>V5QQR2_LEPDE **60 kDa heat shock protein**

MYRLPIAVRSLSLRKAQQISHVQRWYAKDVRFGSEVRALMLQGVDILADAVAVTMGPKGRNVIIEQSWGSPKITK
DGVTVAKGVELKDKFQNIGARLVQDVANNTNEEAGDGTTTATVLARSIAKEGFDNLGKGANPVEIRKGIMLAVEKI
TETLKTLSKPVTTPEEICQVATISANGDTSVGNLIADAMKRVGKEGVITVKDGKTLIDGLEVIEGMKFDRGYISPYFVN
TTKGAKVEYQDALILLSEKKISSVQSIVPALELANAQRKPLIIIAEDVDGEALTTLVVNRLRIGLQVAAVKAPGFGDNRK
ATLTDMAIATGGIVFGDDANIVKLEDIKLSDLGQIGEIAITKDDTLLLKGKGKKEDIDRRSEQIRDQIESTTSEYEREKLQ
ERLARLASGVAVLKVGGSSEVEVNEKKDRVNDALNATRAAVEEGIVPGGGTALLRCTGSLDSIKPANNDQAIGIEIVK
RALKVPCMTIAKNAGVDGAAVVAKVEQQEGDYGFDALNNEYVHMFERGIIDPTKVVRTAIVDASGVASLLTTAEA
VITEIPKEEPAVPSGMGGMGGMGGMGGMGGMM

>G9FQ75_LEPDE **Multifunctional fusion protein**

MLSVCKQTFSASLQRASVRCLGSVVPELKINDFGVKNEPVYEYLKGSKERVELEKELKETAAKTEDVPIIGDKEFRTKD
VRYQVMPHNHKKQIAKFYYADRDLLNKAIETACEAQKKWDMVPIPERLRIWETAASLMASQYRAKLNAATMLGQ
AKTIIQAEIDSAAELIDFFRLNAYFLKEATKYQPISENPKITKNSMRYRGIDGFIAAVSPFNFTAIGGNLAYTPALMGNS
VLWKPSDTALLSNWVIFNICREAGVPPGVVNFVPADGPVFGDTITASPHLAGINFTGSVPTFTRLWKQVGENIHIYK
NFPRLIGECGGKNYHFIHPTADVETVVNGTIRSAFEFCGQKCSACSRMYVPESLWPKIKEGLLAKRAQLKIGDPTDP
QSFTSAVIDDKAFKRIKNYVDHAKKSPNLEIIGGGNLMTVWATSSNRRLSRPKIPRIKLLTEEIFGPVLSIYVYKDNKIKE
TMDLIGTSTSFALTGAIFAQDEKFSKSAVEELKMTAGNFYVNDKSTGSVVGQQPFGGARLSGTNDKAGGPHYVLR
WGNPQAIKETFVPLSEIEYPYMKQ

> A0A2D1QUE3_LEPDE **E1 ubiquitin-activating enzyme**

MDESSNRGLSRPMSSAAIAGSSVDPPAKKRKLETSTLEMANSSGPSRASEIDEGLYSRQLYVLGHDAMRRMAKSDV
LISGLGGLGVEIAKNVILGGVKSVTLHDEVVCTISDLSSQFYQFYISESDVGKNRAEACCKSLSELNNYVPTKAYTGPLD
EQFIKNFSVVVLTTSSRSEQLRISEITHSNNIALIIADTRGLFAQVFCDFGESFTVVDTNGEPTVSAMIADISKDKEGVV
TCIDDTRHGMEDGDYVTFSEVQGMTELNNCEPIKIKVLGPYTFSIGDTTSFGKYERGGNASQVKMPKELHFKPLQES
LLAPEFVITDFAKIDSPQQLHLAFTTIHKYVEKNGRVPKPWSNEDADEFVSLAKSIAVDGSNDTEVNGSLLETFAKVA
AGDLNPMNATIGGVVAQEVMKACSGKFHPIYQWLYFDSIECLPQDRSEITEELAAPSGSRYDGQIAVFGKEFQKKL
AALKYFVVGAGAIGCELLKNFAMMGVGTEGQITVTDMDLIEKSNLNRQFLFRPHNVQQPKSATAAKVIKKMNPEV

NVVAHENRVGPETESIYNDAFFEKLDGVANALDNVDARIYMDRRCVYYRKPLLESGTLGTKGNTQVIVPFLSESYSSS
QDPPEKSIPICTLKNFPNAIEHTLQWARDNFEGLFRQNAENASQYLKDAGFIDRTLKLPGVQPLEVLESVKAALVDER
PKSFEDCIAWARNHWQEQYSNQIRQLLFNFPPDQLTTTGQLFWSGPKRCPEALTFDINNPLHLDYVLAAANLKAQ
VYGIPTNRDRAYIAEVVSKVEVPEFVPKSGVKIAVTDSQMAMNGNNVDLDRVTQIKEELPSVEELNGLCLTPLEFEK
DDDTNFHMDFIVAASNLRAANYKIPPADRHKSKLIAGKIIPAIATTTSVVGGLVCFELYKLTRSMKSLEPFKNGFVNLA
LPFFGFSEPIAAPKMEYCGNEWTLWDRFEVQGEMTLSEFLTYFKEKHNLEITMLSQGVCMLYSFFMAKAKAQERL
GLTMSEVVKKVSKKKLEPHVRALVFELCCNDAEGEDVEVPYVKYNLC

>U6BQX5_LEPDE **Heat shock protein 70a**

MAKAPAVGIDLGTTYSCVGVFQHGKVEIIANDQGNRTTPSYVAFTDTERLIGDAAKNQVAMNPNNTIFDAKRLIGR
RFDDSAVQGDMKHWPFEVINESGKPKIKISYKGEEKTFYPEEVSSMVLTKMKETAEAYLGKTVTNAVITVPAYFNDS
QRQATKDAGTISGLQVLRIINEPTAAAIAYGLDKKGQGERNVLIFDLGGGTFDVSILTIEDGIFEVKSTAGDTHLGGED
FDNRMVNHFVQEFKRKYRKDLTSNKRALRRLRTSCERAKRTLSSSTQASIEIDSLFEGIDFYTSITRARFEELNADLFRS
TMEPVEKSIRDAKMDKSQVHDIVLVGGSTRIPKVQKLLQDFFNGKELNKSINPDEAVAYGAAVQAAILHGDKSEEV
QDLLLLDVTPLSLGIETAGGVMTALIKRNTTIPTKQTQTFTTYSDNQPGVLIQVYEGERAMTKDNNLLGKFELTGIPP
APRGVPQIEVTFDIDANGILNVTAIEKSTNKENKITITNDKGRLSKEDIERMVNDAEKYRGEDEKQKATITAKNLLESY
CFNIKSTMEDDKIKDKISESDKTTVMEKCNEVIAWLDANQLAEKEEYEHKQKELENICNPIITKLYQGAGGAPGGMP
GFPGAGAAPGAGGAAGGPGPTIEEVD

>V5QPM9_LEPDE **Heat shock protein 83**

MPEDTQNGEVETFAFQAEIAQLMSLIINTFYSNKEIFLRELISNSSDALDKIRYQSLTNPSCLDSGKDLHIKIVPNKAEG
TLTLIDTGIGMTKADLVNNLGTIAKSGTKAFMEALQAGADISMIGQFGVGFYSAYLVADKVTVVSKNNDDEQYIWE
SSAGGSFTIRQDHGEPLGRGTKIVLQIKEDQTEFLEENKIKGIVKKHSQFIGYPIKLLVEKEREKELSDDEAEDEKKEGEE
EDKDKPKIEDVGEDEDEDKKEEPKKKKKTIKEKYTEDEELNKTKPIWTRNADDISQEEYGEFYKSLTNDWEDHLAVKH
FSVEGQLEFRALLFVPRRVPFDLFENKKRKNNIKLYVRRVFIMDNCEDLIPEYLNFIKGVVDSEDLPLNISREMLQQNK
ILKVIRKNLVKKCLELFEELSEDKDGYKKFYEQFSKNLKLGIHEDSQNKAKLADLLRFHTSASGDEACSLKDYVSRMKE
GQKHIYIITGESKDQVAHSAFVERVKKRGFEVVYMTEPIDEYVVQQLKEYDGKTLVSVTKEGLELPEDEDEKKKHEED
KTKFESLCKVMKSILDSKVEKVVVSNRLVESPCCIVTSQYGWTANMERIMKAQALRDTSTMGYMAAKKHLEINPD
HSIIENLRQKTEVDKNDKAVKDLVILLFETALLSSGFTLDEPQVHASRIYRMIKLGLGIDEEESMLTEETPAGDAPSADA
GDSEDASRMEEVD

>A0A0E3ISE4_LEPDE **Actin**

MCDDDVAALVVDNGSGMCKAGFAGDDAPRAVFPSIVGRPRHQGVMVGMGQKDSYVGDEAQSKRGILTLKYPIE
HGIITNWDDMEKIWHHTFYNELRVAPEEHPVLLTEAPLNPKANREKMTQIMFETFNTPAMYVAIQAVLSLYASGRT
TGIVLDSGDGVTHTVPIYEGYALPHAILRLDLAGRDLTDYLMKILTERGYSFTTTAEREIVRDIKEKLCYVALDFEQEMA
TAAASTSLEKSYELPDGQVITIGNERFRCPEALFQPSFLGMESSGIHETVYNSIMKCDVDIRKDLYANTVLSGGTTMY
PGIADRMQKEITALAPSTIKIKIIAPPERKYSVWIGGSILASLSTFQQMWISKQEYDESGPGIVHRKCF

>E7CIZ1_LEPDE **Glycoside hydrolase family protein 48**

MLLGVMKTFIVFLVGCVSLINGGEYLNRFTQQYNKIHDAANGYFSPKGIPYHAVETLIVEAPDYGHQTTSEAYSYWL
WLEAMYGAVNGDFSKFTAAWQNMETYMIPNHASQPTNNYYNPGKPATFAPEGDYPNQYPSQMQFNVPVGQD

PIYQELVNTYGTSDVYLMNWLLDVDNKYGFGNQPGQCQLGPSVEGPSFMNSFQRGPQESVWRTIPQTTCDNFRF
GGQNGFLDLFVGDSHYEQQWKYTIASDAEARAIQAAFWALQWAKDKNQQGAVSDTISKASKMGDFLRYAFFDK
YFKKIGNCIGTYACPGGYGKDSAHYLLSWYMAWGGSLYGNWAWRISDFYAHFGYQNPMTAYVMSKVDEFKPRS
PTGVSDWEMSLERQIEFYEYLQSKEGAFAGGATNSYEGRYETPPANLMNNTFHGMWYEWEPVYHNPPSNRWFG
MQAWSTDRLAQYYYVSGDEKVRPLLDKWVNWVLPEISYDANSYKIPEWLDWSGVPPAVDVSIEKYGTDIGSASAL
ARTLSYYAAKTGDQKVKAVAKGLLDGIWNNHQTTKGVAMEEVMDAYSQFNEEVYVPPGWVGHYPDGTEIKAPT
TFIGIRPWYKNDPDWPKVEAYLNGGPAPKFTYHRFWQQCDIAIAQGTYGLLFNE

>A0A0A7ENR4_LEPDE **Carboxylic ester hydrolase**

MNISTLLVLVTTISFAVADILVTIPNGKLRGRKEYSQRGISFYAFQQIPYAKPPVGELRFREPLPPDNWNGILDATYND
KSCIQFSNYYNANISNYENEDCLYINVYTPEFPSTNLSLPVMYFIYGGGFLNGAANFEYAGPHYLLESGVIVVTVNYRV
GAFGFLATGDKIIPGNYGLKDQQMGLKWVQKNIKYFGGDPEKVTIFGQSAGGSSVAFQVMSKGSKGLFRAAIAQS
GSNIVPWAYQRSYKTLAFKIGAALGKPLNEDTDSDKLLAFLRTIPAKQLNTVSTQVYVDNLFIDQMTDGLVFTPVIEP
EHETAFITENMYEAIENGRMTRVPLMIGMCSEEQLGKLSDDYFPTEIQNYDNDITLFVSRNMHITDRNKLIQVGQTI
HDWYLKGRLEDDKAGTIRYLSDASFTRAIIRHAELQSKFSDVYFYEFSYSGQLGGNTGPFIDGAGKVKHSEDLNYIMT
WSNWTGLNNYPKDDILTSDRYRTMLTNFAKYLNPTPEKTSLFQNLIWPKVTPDNFQYLNIDTNLSIQKNPRGEIYQK
WLKIYEENAVRPLDTF

> V9PBG4_LEPDE **Cathepsin B**

MTMNWCLLAGVITLFLSVSSLPYYEDDFSDIQGPYCENIGCCNDRQDSCSVPILGTLCYCDEFCNHTRVDDCCPDF
WYHCKGIPPPTTTTTTTRGPPQPIVGCEVEGSNRTVFWKEKVIVNCNECICESAGRDKVELLCETNVCLMDPMITET
INRNPEKFGWTAANYSEFWGRTLDEGIKLRLGTLPPQQFVMRMNSVRRMYDPNALPREFDSEEIWPGYISGIRDQ
GWCGSSWALSTAAVASDRYAIVSKGKEAVELSAQNLISCDTKGQQSCSGGHLDRAWSFTKYYGLVDEECFPYVGR
NEPCTIKRQGSLARAGCRPPKYGDRKSRYTVGPAYRLGNETDIMYEITKSGPVQATMKVHHDFFTYNGGIYKHSDL
NLNDRHGYHSVRIIGWGEEMTYNGLQKYWKVANSWGPNWGENGYFRIARGTDESEIESFVIASWPGVDRKILLM
AEELPNPA

> A0A290GAZ2_LEPDE **Yellow-x2**

MKSNNFILFFLVGVLSAYAEKFKVTREWKYINFTWPTEEVYRAAEANGAYIPENNIIAGIKHFDDYFYLSLPRMKSGV
PATLARIPAGMTQDTSPLLEPFPSWEMNKLDDCDSLQNVQNVEIDPKGQIWIIDGGRTDTLLNPVSRCPPKLVIFDI
KKNATTTAYTFPNNVANANHSFLYDIVVDDTDSGYAYITDNSGTDPGIVVFSVKDHHSWKLRHSQTMKADPTASA
FKVNGVMINVPLNIAGIALGPRIHKSANRIIVDEDREVFFCPVSSLHLYSINTTVLRNEMNSLNDGEYQGEVKDLGLK
ASQTVGMTMNNRGILYYTLLSTNSIAKWDTHTPFQTRQRIIARDPRFLEWPNSFSFDQSGNITVLVNRLNRFIYDKL
NLNEPNFRLITAYVGGKSYMYDQGYDYTVDPNATVTTTTENADLPQPEILPGSDNDPYLIPQPLPERKSESEPAPETT
PVPEPEPKPEPNSEPVPEPKPEPNSEPEPEPKAEPEPTAEPEPEPKGEHVNQEQANHDDMSTDHDHMNHDNTME
DSKPANQSSVLIQEPVAVESSSSKLVCTLIGVFTACLLFAF

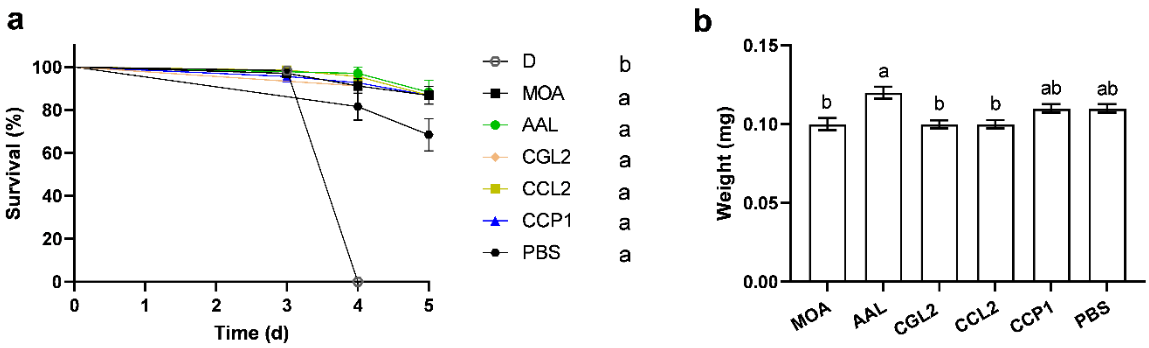

Figure S8 Feeding trials of *A. mellifera* larvae. The following lectins were used: MOA, AAL, CGL2, CCL2, and CCP1 (all at 1 mg/mL). Dimethoate (D) and phosphate-buffered saline (PBS) were used as positive and negative controls, respectively. The trials lasted for 5 days. (a) Survival curves and (b) weight changes of *A. mellifera*. Different lowercase letters indicate significant differences ($P < 0.05$) between treatments, assessed with the Benjamini-Hochberg (a) and Games-Howell post hoc tests (b).

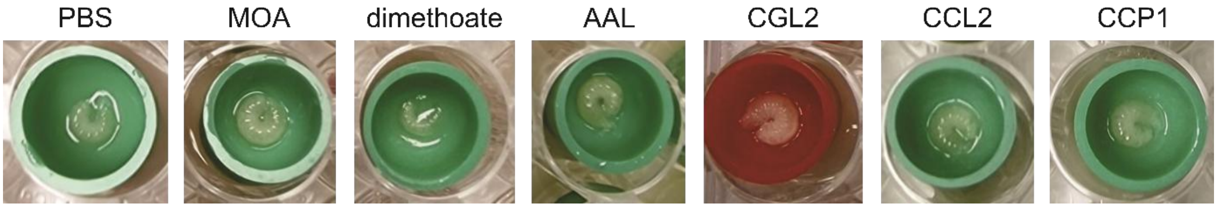

Figure S9 Representative images of honey bee larvae on the third day of feeding trials with selected lectins at 1 mg/mL and phosphate-buffered saline (PBS) as a negative control, and dimethoate insecticide as a positive control.

REFERENCES

Gupta R, Brunak S. Prediction of glycosylation across the human proteome and the correlation to protein function. Pac Symp Biocomput. 2002:310-22.

Steentoft C, Vakhrushev SY, Joshi HJ, Kong Y, Vester-Christensen MB, Schjoldager KT, Lavrsen K, Dabelsteen S, Pedersen NB, Marcos-Silva L, Gupta R, Bennett EP, Mandel U, Brunak S, Wandall HH, Levery SB, Clausen H. Precision mapping of the human O-GalNAc glycoproteome through SimpleCell technology. EMBO J. 2013 May 15;32(10):1478-88. doi: 10.1038/emboj.2013.79.

Wohlschlager T, Butschi A, Zurfluh K, Vonesch SC, Auf dem Keller U, Gehrig P, Bleuler-Martinez S, Hengartner MO, Aebi M, Künzler M. Nematotoxicity of *Marasmius oreades* agglutinin (MOA) depends on glycolipid binding and cysteine protease activity. J Biol Chem. 2011 Sep 2;286(35):30337-30343. doi: 10.1074/jbc.M111.258202.
